# Factory benefits to paying workers more: The critical role of compensation systems in apparel manufacturing

**DOI:** 10.1371/journal.pone.0227510

**Published:** 2020-02-05

**Authors:** Niklas Lollo, Dara O’Rourke

**Affiliations:** 1 Energy & Resources Group, University of California, Berkeley, Berkeley, California, United States of America; 2 Department of Environmental Science, Policy, and Management, University of California, Berkeley, California, United States of America; Xiamen University, CHINA

## Abstract

While many stakeholders believe worker wages in global supply chains are too low, there is disagreement about what, if anything, can be done to raise wages. Through a two-year quasi-experiment in an operating apparel factory, we assess the effects on productivity and profits of raising worker wages with a re-designed compensation system. We show that, even within current factory margins and constraints, important wage gains (4.2–9.7%) are possible and profitable. Productivity increased 8–10%-points while turnover decreased markedly. Workers were motivated by the potential for increased wages from an accelerating group rate as well as increased engagement and sense of fair compensation. Workers focused their increased effort on reducing quality defects and tardiness, two behaviors which individual workers largely control. Additional productivity-increasing behaviors were constrained by skill, position, and conflicts arising from free riders. Advanced apparel manufacturing demands a more engaged workforce; this research provides early evidence that compensation systems can be a critical tool to meet multiple needs.

## Introduction

Factory worker pay in global apparel supply chains remains a contentious issue [1,2]. In an industry focused on continuously-increasing efficiency and cost-reductions to meet consumer demands, worker wages have often been squeezed to meet brand, retailer, and supplier profit targets [3,4]. Despite increasing productivity, factory workers have experienced flat or declining real wages around the world [5,6].

While many stakeholders believe worker wages are too low, there is disagreement about what, if anything, can or should be done. Global brands assert that they have limited ability to increase the wages paid by contracted factories, that their suppliers pay “prevailing wages”, and that they have little influence over all-important, government-set minimum wages [3,7,8]. Factory managers argue that their margins are too thin and their contracting relationships are too tenuous, among other rationales, to pay workers more [8]. Workers, unions, and NGOs, on the other hand, advocate for a “Living Wage” and assert that brand margins and consumer budgets can accommodate wage increases [9–12], and that it can be profitable for factories [13] and beneficial for economic development [14].

Amidst this longstanding struggle for better working conditions (with wages being one of the most significant issues), the apparel industry is also undergoing rapid structural changes. Known colloquially as “fast fashion”, the market is demanding increasing speed-to-market and product differentiation [15,16]. As a result, factory goals have shifted from total output to the continuous flow of high-quality, in-demand styles. At the request of buyers, manufacturers have adopted “Lean” production principles—continuously increasing productivity and quality while eliminating waste—to modernize their factory layouts, machinery, and IT systems [17,18].

These Lean changes to the factory floor have compelled complementary managerial and human resource (HR) policies [19]. It is more common now for factory floor workers to be trained in multiple skills and asked to identify and solve problems without depending on hierarchical decision-making [20–22]. Despite these and other advances in HR practices, many Lean factories still employ similar compensation systems as they did 40 years ago [6]. And very few factories align compensation with modern job expectations and factory goals [23].

While firm practices can be considered *prima facie* evidence for efficient and effective policy [24](such that sweating workers may actually be a perverse, profit-maximizing strategy), apparel factory management practices are likely to be sub-optimal for a number of reasons [25]. One major contributing factor is that though women constitute a majority of apparel factory workers, they rarely occupy management or high-status positions [26,27]. This stark gendering of positions often contributes to antiquated, gender-biased wage discrimination. Other causes of inefficient policy include that managers may lack knowledge (about what workers want, the ideal policy, the benefits afforded by said policy, and so on), skills, or attentional capacity [28–30]. Instead, management practices may simply correspond to local norms and historical practices (bringing a certain inertia).

Moreover, whether these biases are present or not, as with any business, changing external conditions merit re-examination of firm-level practices. We believe compensation systems—which may already be inefficient—can play a critical role in responding effectively to both the increasing normative pressure to raise worker wages and to meet the new production demands of fast fashion.

Our paper addresses the question: How and under what conditions can wage increases be structured to improve productivity and profitability within an agile and competitive factory environment? Through a two-year quasi-experiment in an operating apparel factory, we assess the effects on productivity and profits of raising worker wages with a re-designed compensation system. We show that, even within current factory margins and constraints, important wage gains (4.2–9.7%) are possible and profitable. Under the treatments, productivity increased 8–10%-points while turnover decreased markedly. We next summarize the relevant literature on compensation to help explain our results.

### Motivating workers through wages

The classic way to incent factory workers is by the “piece rate”, which pays workers according to their marginal productivity [31,32]. Because apparel factory work—unlike agricultural picking, a commonly-studied job in wage design—is team-based, most apparel workers are paid according to group piece-rates (what we call a “group rate”) [23,33,34]. For a review of literature on improved incentives in other manufacturing contexts, see [33]. Studies such as Boning et al. (2007) and Hamilton et al. (2003) have examined the effect of incentives in US manufacturing, while Blasi et al. (2009) conducted a review of studies on workplace incentive systems.

Workers, on the other hand, regulate their behavior in order to optimize between wages received and effort exerted [35,36]. Individuals are not simply motivated by ever higher wages; they have a “reference” wage determined by their marginal utility of income [37–39]. Assuming no significant change in preferences, this is essentially the wage workers would be satisfied by. If workers are receiving their reference wage, then a wage increase is likely to have no effect, or possibly a negative effect, on productivity [37]. All else equal, once the reference wage is obtained, we should begin to observe diminishing increases in effort, if not compensatory decreases in effort [36]. If living wage advocates are correct, worker wages are currently much lower than their reference wages, and thus wage increases should motivate increases in effort simply because workers want to earn more income.

There are many reasons to believe that the biggest problem with group rates is that they are set too low for workers to earn satisfactory wages. In the apparel industry, worker wages are *de facto* set by national minimum-wage policy [8]. Factory management, required to pay the minimum wage, often sets the group rate such that workers will achieve the minimum wage when a target output is reached. The goal for workers is of course to beat the target output. However, modern manufacturing demands makes that goal increasingly challenging and frustrating. Target output is determined by the “standard allowable minutes” (SAM) of a given garment and the available working minutes of the production line. There are general, “universal” SAMs calculated by third-party organizations using industrial engineering techniques (e.g., time-measurements of ideal physical movement), though the SAM for each given garment is set through brand-factory negotiation. With downward pressure on the SAM due to brand power, beating the SAM (and thus the target output) becomes harder over time.

The standard practice of “pay for time” compensation, while dominant and presumably efficient, becomes problematic because the difference between the minimum wage and pay-for-performance is minimized. Workers are not motivated to put in additional effort beyond the minimum required to avoid punishment. They are also more likely to leave the job altogether [40]. On days with new product-styles, more difficult product-styles, or multiple product-style changes (requiring more or fewer workers and/or different machines), simply working for overtime (when pay increases by time-and-one-half) becomes the best method to maximize take-home pay while minimizing effort. Even though it costs the factory more per labor hour, managers accept overtime as normal business practice, as it allows them to accommodate variable order sizes, hire fewer full-time workers, flex worker hours during production peaks, and not have to lay off workers during slow periods [25,41].

Incentivizing increases in productivity through increasing compensation is likely to fail if several other conditions are not met. First, workers need to trust management. If workers believe that the group rate (or SAMs) may be subsequently lowered once they demonstrate higher productivity, workers may avoid increasing their effort [33]. Second, workers need to perceive the expected wages as fair given the effort to obtain them [35,36]. Third, workers need to have control over processes that would increase productivity [42–45]. Fourth, problems arising from the free rider issue inherent to group rates—every worker receives the same wage, which can lead to higher-skilled or harder-working workers becoming de-motivated—can be significant [23,34,46,47]. A better team culture and/or skill and seniority bonuses can help minimize the issue. However, a recent assessment suggests skill and seniority bonuses are uncommon [6]. All told, wage increases should motivate workers to increase productivity if they are currently below their reference wage, trust management that wage gains are permanent, the wage gains are fair given the effort required to obtain them, there are productivity-increasing actions workers have control over, and conflicts from free rider issues are not significant.

### Project summary

**Fig 1:**
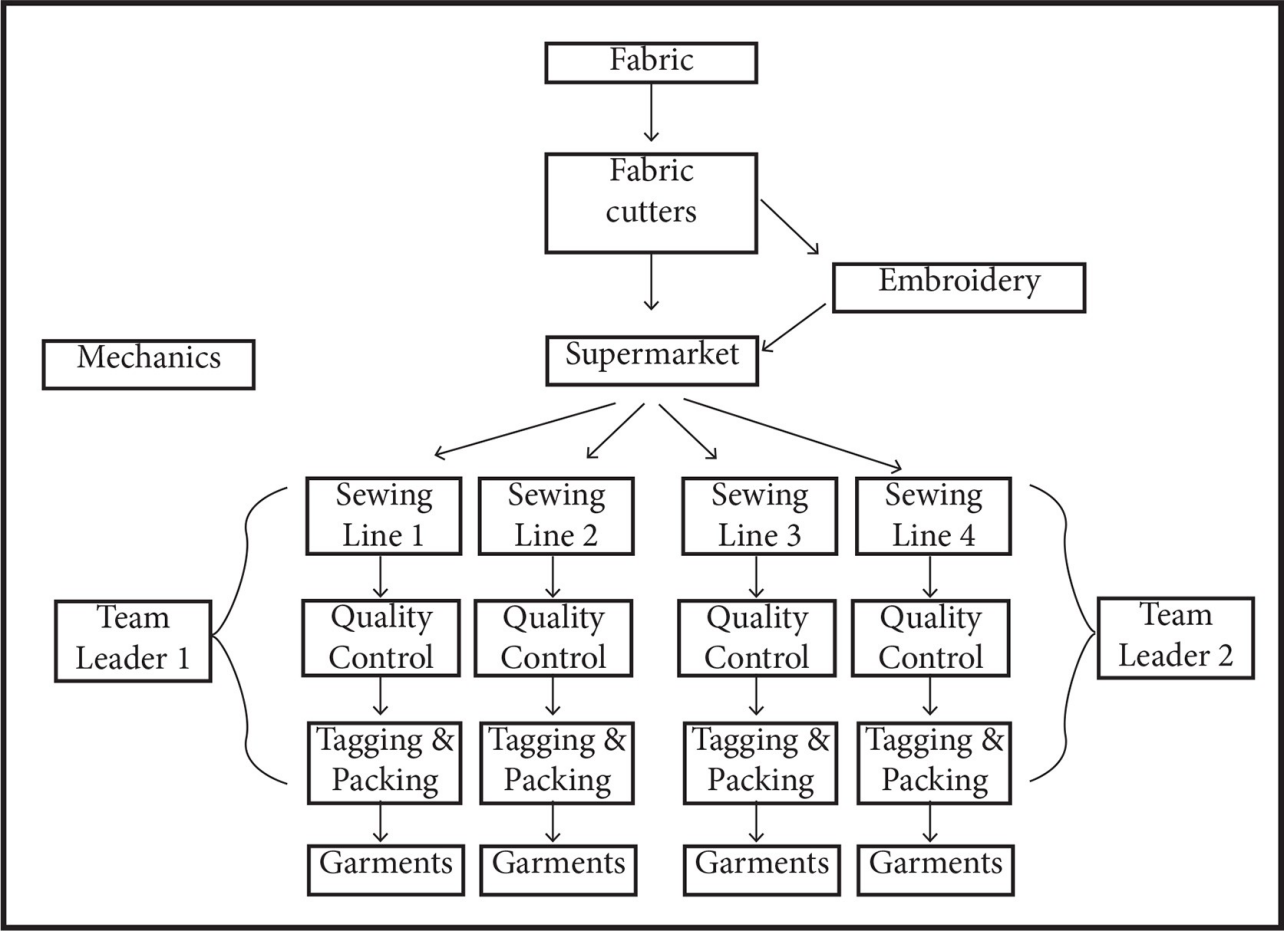
The production schematic for one business unit. In the factory, the fabric cutters, embroidery, and supermarket supply to multiple lines. Each team leader oversees two lines. Mechanics help fix machines anywhere along this process. At the end of each line, there was an LCD screen that displayed information about the target output and current production level.

In a two-year quasi-experiment in Thailand, we made three specific changes to the operating apparel factory’s existing compensation system on eight production lines (that is, six treatment lines, plus two control lines out of 23 total lines). First, an accelerating group-rate wage structure was introduced to the production line. We tested three different versions of this accelerating group-rate (two lines per version; two lines were held as control). The compensation systems can be described as: 1) accelerating group-rate (“Productivity”); 2) accelerating group-rate focused on Productivity with additional Quality and Waste Reduction incentives (“PQWR”); and 3) accelerating group-rate that allowed workers to elect to go home when they reached a pre-specified target wage (“Target wage”). Second, these wage structures were applied to all workers in the relevant production chain (cutters, line managers, supermarket, etc.; see Fig 1 for more graphic detail), and monthly and yearly bonuses were removed where those had been applied (mostly for lower-level managers). Third, new communication procedures around compensation were introduced via LCD monitors and team meetings.

Employing a difference-in-difference identification strategy with a two-year panel dataset, we find the PQWR and Target Wage treatments increased productivity about 8%-points and 10%-points, respectively, while the Productivity treatment saw no effect. These gains were obtained in the short-term and persisted, ruling out a mere novelty effect. The treatments raised wages between 20 and 46 baht per ten-hour day from a pre-treatment average of approximately 480 baht. These wage increases of between 4.2% and 9.7%, raised wages much closer to the estimated living wage of 606 baht in 2017, and to workers self-reported-preferred wage of 700 baht. Workers, in four sets of focus groups conducted by a third-party, reported increased satisfaction with the new wages, and more satisfaction with management, as a result. Turnover decreased in the PQWR and Target wage treatment groups, and workers stated they more motivated by the increased potential wage, and that it created a starker difference with the “pay for time” option.

Newly motivated workers focused their increased effort on behaviors for which they had the most control. The PQWR and Target wage treatments raised the quality rate by about 1.8%-points, while Productivity had no effect. While stress did not appear to increase, we find worker tardiness decreased. Yet additional productive behaviors which required more effort and stress, such as supporting team members and line balancing, were largely unaffected. This can be explained by the still-present free-rider issues; workers continually noted their frustration with lower-skilled team members. Similarly, line workers lacked the skills and authority to remove some of the more consequential constraints on productivity, such as material and machine downtime. The incentive for those workers who were responsible for those issues was found to be less effective.

For the factory, profits increased as output gains outweighed profit-per-garment losses on five of the six treatment lines. Moreover, the decreased turnover resulted in significant avoided costs. The clearest testament to the intervention’s success is that management expanded parts of the new compensation framework—the accelerating wage structure and production chain alignment—across the entire factory as soon as the quasi-experiment ended.

This research suggests that, even within current factory constraints, important worker wage gains are possible and profitable. Management should consider improving compensation as a means to retain workers and incent better performance. The results should be of interest to industry stakeholders and to scholars of worker compensation regarding the effects of raising wages on firm outcomes. The paper is structured as follows. The next section provides details on the quasi-experiment. The following section covers results and discussion. We then conclude.

## Quasi-experimental details

### Factory context

In 2015, we partnered with a multinational company and one of the company’s strategic suppliers—a manufacturer for the company’s more innovative and high-value products—based in Thailand. Of the supplier’s facilities, we selected a “cut-and-sew” final-assembly factory for the intervention. The factory workforce was entirely ethnic Thai. The gender balance was typical of apparel manufacturing: about 95% of the sewing line workers, quality control personnel, line managers, and supervisors were female, while all of the mechanics and upper management were men.

The factory had fast-fashion production characteristics: continuously changing product styles, small-batch orders, high-pressure to meet on-time delivery targets, and a competitive price negotiation process. To meet these demands, the factory, over the preceding years, had implemented many features of a modern Lean production system, from the physical factory layout to advanced technology and machinery, all to encourage more efficient production flow.

In addition, between May and November 2015, the factory management had begun to implement new human resource policies, including management training. For workers, there was an emphasis on skills and engagement. First, all sewing line team members and supervisors received five trainings, from technical to interpersonal skills. Second, the management instituted a social dialogue system with worker election of line representatives, training of line representatives on how to hold meetings, elicit input, elevate issues to management, and communicate resolutions (without supervisors present). Third, management trained workers and supervisors in stress management and provided time each morning for a short set of stress management activities. And finally, management supported discrete 5-day, worker-only problem-solving events—“Kaizens”—in which workers would raise issues (perhaps using insights from an internal “engagement and well-being” survey), develop solutions, and then present the ideas to management.

Before the new HR program, worker dissatisfaction was quite high, as measured in an internal engagement and well-being survey. Initial focus groups revealed that line managers were highly dissatisfied as a result of perceived undue pressure from both upper management and sewing line workers, along with unsatisfactory compensation. Sewing line workers, in a separate focus group, expressed frustration with nearly all other workers—quality control personnel, mechanics, and line managers—for lack of support. Along with unsatisfying compensation, these workplace tensions were contributing to turnover rates upwards of 80% per year on some lines.

To understand how these workplace tensions affected productivity, first we need to understand how a garment is produced. Fabric enters the facility and is cut into pieces of a given garment. These pieces are bundled and sent to sewing lines in another part of the factory. Once the garment is assembled and has passed the quality inspection, the garments are tagged, packed, and boxed for shipment. Fig 1 presents a basic schematic of the production process for one business unit involving four sewing lines.

Production lines are generally assigned the number of workers that match the number of discrete operations a garment requires, typically about 20. These workers form a team and generally stay on their given line. However, workers may be added (from a “relief” team) or removed from lines on any given day as line managers try to balance production as product-styles change during the day, workers are absent, or bottlenecks emerge. How effectively this “line balancing” is performed has large consequences for productivity.

Despite being a central worker concern, wages had not been addressed by factory management within the new HR program. Worker compensation and incentives varied by position. Sewing line workers, whose pre-existing compensation was the starting point for the intervention, received the greater of the minimum wage or a group rate multiplied by total line output. Over the entirety of the study period, the Thai minimum wage was 300 baht over 8 hours and 1.5 times the hourly rate for overtime. For reference, the exchange rate between Thai baht and USD was approximately 35 baht to one USD. Workers typically worked 8 hours on Monday and Saturday, and 11 hours on Tuesday through Friday, for a total of 60 weekly hours, which is the maximum hours allowed under Thai law. Line managers were paid a monthly salary with uncertain profit-based bonuses. Quality control personnel received the minimum wage plus an incentive for identifying product-defects. Mechanics, whose efforts to fix machines were critical to achieving production flow, were paid through a separate compensation framework. Fabric cutters and workers that transported the garment bundles simply received the minimum wage. There were no seniority or skill-based bonuses in the factory.

Line workers, before the intervention, were making 480 baht for a 10-hour day. This was slightly above the 412.5 baht minimum required by law for ten hours. Though Thailand lacked a Living Wage estimate produced by the ILO-Anker method, the Asia Floor Wage, a second-best option, was estimated in 2015 at 13,359 Baht per month and in 2017 at 15,140 Baht per month (https://asia.floorwage.org/what. Accessed July 8^th^, 2019). Assuming 25 workdays per month, these figures translate to 534- and 606-baht per day. With consistent year-over-year change, at the onset of the intervention in 2016, the Asia Floor Wage would have been 570 baht per day, leaving a daily wage gap of 90-baht per line-worker. Workers stated in focus groups they that 700 baht per day would be “good”. This would mean line workers had a 220-baht daily wage gap.

### Quasi-experimental design

We evaluated three compensation system treatments with eight sewing lines—six treatment lines and two control lines—out of 23 lines in the factory. Data collection began one year prior to the intervention and continued for one year following the intervention (from January 1, 2015 until January 15, 2017) to control for seasonality of order volume and holidays. The primary data is daily human resource and productivity data for each of the eight sewing lines. Each observation represents one line-day. Factory management also provided demographic information and monthly turnover data, while a third-party conducted four sets of focus groups with workers before, during, and after the intervention. Informed consent was obtained verbally by workers and managers for the general study as well as specifically for those workers who participated in focus groups. This study, including verbal consent, was approved by the UC Berkeley Committee for Protection of Human Subjects, Protocol ID 2015-09-7924. Verbal consent without record was deemed acceptable according to UCB IRB reasoning: “The research presents no more than minimal risk of harm to subjects AND involves no procedures for which written consent is normally required outside of the research context.”

We employed a quasi-experimental design. The lines were not randomly selected; the company and factory management selected what they considered to be four average pairs of lines. Since the line manager is critical for performance [48], and line managers oversaw two lines each, line-pairs were evaluated against one another. Using data from the prior year (2014–2015), the company and factory management tried to control for line-level variables they thought would influence the study: productivity, number of workers, worker skill, location in the factory, the physical line shape, and line manager capability. Workers were not able to self-select onto or off of lines that received a treatment. The other 15 lines in the factory were not monitored during our intervention, but were likely aware of the intervention and present a possible source of bias.

The intervention aimed to increase wages through an accelerating group rate [49]. The control group continued to be compensated as had been previously done: they received the greater of the minimum wage or a group rate multiplied by total line output. For the treatment, however, the group rate would increase as certain productivity targets were reached. Productivity, a measure of labor-time efficiency, can be expressed as:

$$Productivity=\frac{Output*\frac{SAM}{garment}}{\#of\;workers*working\;minutes}$$ (1)

Through analysis of historical data, we conservatively estimated a breakeven-point for the factory at 85% productivity. So, at 90% productivity, the group-rate for all garments produced that day (even on days with multiple styles) was multiplied by 1.06. This multiplier increased by 0.06 for every five-percentage-point productivity increment thereafter, with a ceiling at 1.48 for 125% productivity. This new wage structure constituted the first treatment, which was called “Productivity”, and served as the base for the other two treatments. According to the new incentive structure, all eight lines would have received some extra incentive 60% of the days during the pre-treatment period.

The second treatment, “Productivity, Quality, and Waste Reduction” (PQWR), used this same new wage calculation, but sought to anticipate and avoid a tradeoff between productivity and quality. Since inefficiencies can cascade across the production chain, workers at each stage were incented to maintain high-quality standards and to reduce material waste. For instance, fabric cutters received a 10-baht bonus when they identified that a fabric layout could be remarked to optimize fabric usage. For sewing line workers, there was a 20-baht bonus if the line achieved a 97% quality rate for the day. The quality rate is the number of garments that did not need re-work nor were defective divided by the total output. Initially, workers had five baht deducted if quality rate went below 95% and received a 10 baht increase above 99%. In June of 2016, the factory removed the deduction and set the positive incentive at 97% quality rate. While not ideal from an experimental point-of-view, this shows that worker feedback was taken seriously; workers did not like the deduction.

The third treatment, “Target wage,” used the same new wage calculation as the Productivity treatment, but if the line reached a given target wage, then they could elect to go home for the day. After consulting with management and workers and analyzing the previous wage data, the target wage was set at 500 baht in 8 hours and 650 baht in 10 hours. This target wage effectively aimed to codify the reference wage. The Target wage treatment began one month later on January 15, 2016.

Each treatment also received several complementary measures: incentive alignment and better wage communication. These changes were made in concert with the compensation formula change, so we did not test individual effects, nor, of course, are the effects independent of the factory’s pre-existing HR practices. First, all workers along the production process—sewing line workers, team leaders, supervisors, mechanics, quality control personnel, and cutting room team members associated with two of the treatment lines—would be compensated according to the line’s productivity. Workers associated with multiple lines, like team leaders and fabric cutters, were compensated based on an average of associated lines. For all workers, the new wage was either an upgrade in pay or maintained pay at a similar level. Workers were guaranteed to not be lower than they would have been under the old system. Biweekly paychecks showed workers their wage alongside what they would have received under the previous compensation system (for positions without profit-sharing bonuses).

Second, the factory reconfigured pre-existing LCD screens with more detailed hourly wage, target output, hourly rates of productivity, and expected wage information for each treatment line. Workers were also trained to interpret the reconfigured LCD screens, in order to help workers to make informed, real-time decisions about their effort based on expected wages. Through focus groups, workers recommended and it was adopted that the LCD screens would display the number of garments needed to reach the next incentive bracket, rather than show a percentage measure of productivity.

In the focus groups conducted by a third-party consultant, workers were introduced to the new wages. Worker feedback from these focus groups informed the final wage targets, productivity levels, and quality bonuses. Workers also asked for mid-level management to personally explain the new wage system to all workers in the treatment. This request was granted.

### Empirical strategy

Establishing causality with a quasi-experimental design presents challenges [50]. To estimate the treatment effect, we use the difference-in-difference strategy with an ordinary least squares (OLS) regression model with fixed effects. We bolster this analysis with turnover data and focus groups.

In each of four focus group sessions—one before the treatment, two during, and one after the intervention—a trained, local facilitator separately queried line members, line managers, quality control personnel, higher-level managers, and mechanics. The consultant worked with factory mid-level management to select workers. Focus groups included a few team members from each treatment group, as well as control lines. The resulting documents were analyzed thematically, looking for patterns and deviations from the different focus group members. The focus groups provided important information about worker perception of wages and about team dynamics across treatments and over time.

The primary line-level productivity and wage dataset contains over 4000 observations with full and consistent data (i.e., no outliers or missing data). One observation entails one line-day, with data on productivity, output, number of workers, style ID, and so on. Observations are at the line-level, not worker-level. The observations that are missing (about 200 line-days were missing data on either the dependent or independent variables) do not disproportionately fall on any one line or treatment group. Table 1 below shows descriptive statistics of the key variables. The Supporting Information contains summary statistics grouped by treatment (see Tables A-D in S1 Appendix).

**Table 1:** Descriptive statistics of all variables over the full period (January 2015-January 2017).

Statistic	N	Mean	St. Dev.	Min	Pctl(25)	Pctl(75)	Max
**Productivity (%)**	4,430	98.08	35.09	2	74	123	178
**Hourly Wage (THB)**	4,425	50.47	10.9	37.5	42.61	56.97	111.49
**Working Hours**	4,425	9.97	1.46	8	8	11	12.5
**Overtime (0,1)**	4,430	0.52	0.5	0	0	1	1
**Number of Workers**	4,430	20.04	1.9	9	19	21	28
**SAM (min/garment)**	4,410	22.69	6.28	0	19.86	26.35	78
**Lead Time (days)**	4,404	6.66	7.25	1	2	9	57
**Style Familiarity (style-days/line)**	4,430	54.09	47.95	1	17	81	179
**Tardiness (min)**	4,423	4.93	19.66	0	0	4	725
**Unplanned Absente**	4,430	1.76	3.66	0	0	4.2	77

The general regression model specification is:

$$y_{it}=\alpha_{it}+\beta_{it}X_{it}+\delta_{it}W_{it}+\gamma_{it}Z_{it}+\varepsilon_{it}$$ (2)

Where *y_it_* is the outcome variable, indexed by line (*i*) and time (*t*); *X_it_* is the treatment dummy variable (0,1); *W_it_* is a vector of covariates; *Z_it_* is a vector of fixed effects; and, *ε_it_* is the error term, and is assumed E[*ε_it_*|*X,W,Z*] = 0.

The treatment variable, *X_it_*, can be decomposed into its interaction terms:

$$X_{it}=phase_{it}*treat_{it}$$ (3)

Where, *phase_it_* is a (0,1) dummy for the intervention period, with a value of one during the intervention, and *treat_it_* is a (0,1) dummy variable for treatment, with a value of one if a line received a treatment. Our coefficient of interest in Eq 2, *β_it_*, therefore, captures the mean difference between treatment and control groups, controlling for any pre-treatment differences by group.

A difference-in-difference strategy depends on the parallel trends assumption [50]. Despite non-random line selection, the parallel trends assumption was not violated in all cases when controls (line, style, and number of workers) are included. The parallel trend assumption did not hold for a simple time trend, likely due to strong seasonality and differences in style consistency (see Table E in S1 Appendix for the results from the two-tailed t-test; see Fig A in S1 Appendix for a plot of the time trend using regression residuals).

Using a January 2016 snapshot of demographic data, we did not find significant differences in worker age or length of employment. There were some gender composition differences between the lines (see Tables F-H in S1 Appendix). And, when comparing line manager characteristics—years at the facility, years as a manager, and number of trainings—the only significant difference was that the PQWR treatment manager had less management experience (as shown in Table I in S1 Appendix). We believe the comparison is still valid, but the point estimates may not be precise.

We estimate treatment effects on productivity, wages, tardiness, quality rate, and factory profit-per-garment. The covariates, whose inclusion vary across regression model specification, include number of workers, work hours, a binary variable for overtime, the number of consecutive days a style has been in production on the line, the expected standard allowable minutes (SAM) to produce the garment, total tardiness in minutes, and unplanned absenteeism. We tested various functional forms for these covariates. The fixed effects, included in each model specification, were garment style, date, and line. Style captures major differences between the 170 styles not accounted for by SAM. Date controls for any temporal variations such as temperature [51], type of garments, and number of orders. Line captures unobservable differences across lines that may cause different outcomes such as the team culture.

We corrected for standard errors using the Newey-West variance estimator [52]. When clustering standard errors at the line level, the general statistical story remained consistent, however the Newey-West method resulted in a better distribution between fitted values and residuals. For more information on data analysis, see the Supporting Information. The regression tables were generated using the “Stargazer” package in R [53].

## Results & discussion

This section proceeds as follows. First, we determine if and by how much the treatment influenced productivity, the central measure of treatment success. We then look for evidence that the treatment had its intended effects: that wage increases, and other treatment features, occurred. By examining if and how workers perceived the wage increases, we explore mechanisms for changes in productivity. Then we look at how worker behavior changed, by examining turnover, absenteeism, tardiness, quality, teamwork, and effort. Finally, we assess the intervention’s effects on firm outcomes including profitability.

### Impact on productivity

**Fig 2:**
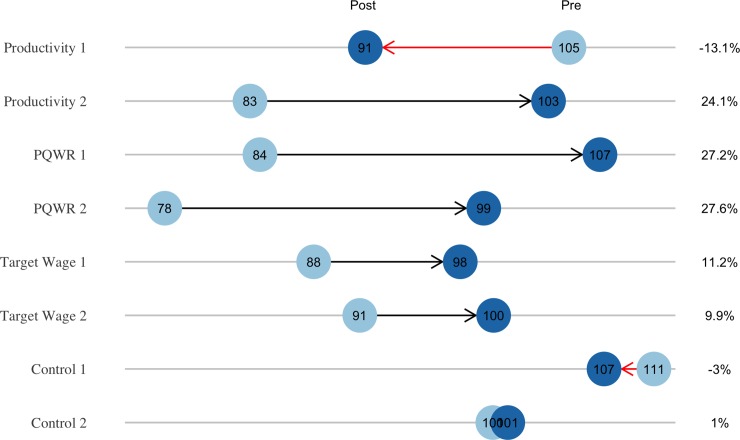
Change in daily productivity between pre-treatment and post-treatment periods for each line. The percentage-points gained or lost is presented on the right-hand side. “1” and “2” denote the line.

Across six OLS specifications presented in Table 2, we find the PQWR and Target wage treatments had positive, significant effects on productivity—10%-points and 8%-points, respectively. Both point estimates increase by about 4%-points when the covariates are removed. We do not find a significant effect for the Productivity treatment. When examining Fig 2, which shows the actual change in productivity between the two periods, we see that only one line in the Productivity treatment appears to be unsuccessful, a discrepancy we explore in detail during our investigation of mechanisms.

**Table 2:** OLS regressions with productivity as the dependent variable.

	*Dependent variable*:					
	Productivity (%)					
	1	2	3	4	5	6
**Productivity treatment**	-2.128	-1.767	-1.861	-2.827	-3.886	-3.692
	(3.805)	(3.841)	(3.289)	(3.429)	(3.497)	(3.518)
**PQWR treatment**	12.075**	12.231**	9.353*	8.611*	8.276*	8.178*
	(5.177)	(5.178)	(4.924)	(4.940)	(4.941)	(4.959)
**Target wage treatment**	13.985***	14.100***	11.696**	10.313**	9.745**	10.240**
	(4.493)	(4.502)	(4.589)	(4.480)	(4.420)	(4.382)
**Overtime**		3.897**	3.975**	4.049**	4.099**	7.590*
		(1.884)	(1.836)	(1.848)	(1.856)	(4.143)
**Familiarity**			0.118***	0.120***	0.118***	0.114***
			(0.025)	(0.025)	(0.024)	(0.025)
**Number of line workers**				38.888	43.541	41.722
				(39.509)	(39.319)	(39.544)
**Square of Number of line workers**				-53.458*	-49.571	-48.214
				(31.837)	(31.817)	(31.639)
**Cube of Number of line workers**				-80.781***	-75.140***	-74.497***
				(23.358)	(24.083)	(23.657)
**SAM**					1.287**	1.290**
					(0.504)	(0.510)
**Work hours**						-1.352
						(1.268)
**Style—consecutive days in production**						0.122
						(0.121)
**Tardiness (minutes)**						-0.015
						(0.020)
**Unplanned absenteeism (%)**						-0.023
						(0.143)
**Observations**	4,372	4,372	4,372	4,372	4,372	4,372

Note

*p<0.1

**p<0.05

***p<0.01

Standard errors corrected for serial correlation using Newey-West variance estimator.

Fixed effects for each specification are date, style, and line.

There is a strong non-linear relationship between work hours and productivity. We would expect effort and productivity to linearly decrease with work hours, however there is no evidence for this effect. Instead, productivity increases significantly with overtime, which may be a result of workers applying greater effort when they can receive higher wages. As we would expect, familiarity is a driver of increased productivity as workers become more adept at making a particular style. In the focus groups, workers consistently said that “longer orders” were better for productivity. Interestingly, SAM has a strong, positive relationship to productivity, which may be a feature of higher SAMs having more leniency baked in (or are harder to project), or that workers are more familiar generally with higher SAMs and this result is capturing that effect. We expect it to be the latter, considering a majority of SAMs were within the 25–40 minute range. Specification 6 includes two endogenous variables—Tardiness and Unplanned absenteeism—so the estimates are likely biased and any interpretation of that specification’s results should be cautious.

### Wages & behavior

Our primary hypothesis was that an increase in wages would yield an increase in productivity. Yet if workers had previously been producing with maximum effort and efficiency, a wage increase would not induce any productivity increase. Moreover, if wages already equaled the reference wage, we might expect a rate increase to yield a decrease in productivity while wages stayed consistent.

As with productivity, we find positive and significant treatment effects on hourly wages, as shown in Table 3 below. For the Productivity treatment, despite no corresponding increase in productivity, hourly wages still increased by about two baht. For the other two treatments, their productivity increase, coupled with the accelerating group rate, raised hourly wages between 3.7 and 4.6 baht per hour. So, on an average ten-hour day, the treatment raised wages between 20 and 46 baht, amounting to a 4.2% and 9.7% average wage increase over the pre-treatment period.

**Table 3:** OLS regressions with hourly wage as the dependent variable.

	*Dependent variable*:					
	Hourly wage (baht)					
	1	2	3	4	5	6
**Productivity treatment**	2.202*	2.597**	2.562**	2.246**	2.115*	2.120*
	(1.281)	(1.298)	(1.078)	(1.083)	(1.095)	(1.095)
**PQWR treatment**	4.986***	5.158***	4.067***	3.831***	3.790***	3.739***
	(1.490)	(1.489)	(1.425)	(1.407)	(1.402)	(1.402)
**Target wage treatment**	5.845***	5.971***	5.059***	4.619**	4.549**	4.600**
	(1.874)	(1.867)	(1.880)	(1.842)	(1.830)	(1.824)
**Overtime**		4.272***	4.301***	4.322***	4.328***	3.021**
		(0.675)	(0.654)	(0.655)	(0.656)	(1.265)
**Familiarity**			0.045***	0.045***	0.045***	0.044***
			(0.008)	(0.008)	(0.008)	(0.008)
**Number of line workers**				15.069	15.645	16.048
				(12.589)	(12.525)	(12.741)
**Square of Number of line workers**				-18.452*	-17.971*	-18.057*
				(9.817)	(9.663)	(9.639)
**Cube of Number of line workers**				-21.799***	-21.101***	-21.315***
				(7.398)	(7.244)	(7.260)
**Work hours**					0.159**	0.155**
					(0.065)	(0.066)
**SAM**						0.505
						(0.406)
**Style—consecutive days in production**						0.021
						(0.034)
**Tardiness (minutes)**						-0.008
						(0.007)
**Unplanned absenteeism (%)**						0.038
						(0.032)
**Observations**	4,372	4,372	4,372	4,372	4,372	4,372

Note

*p<0.1

**p<0.05

***p<0.01

Standard errors corrected for serial correlation using Newey-West variance estimator.

Fixed effects for each specification (1–6) are date, style, and line.

Because the Productivity group received higher wages but did not see corresponding productivity gains, it would suggest there are certain conditions necessary for wage gains to be effective motivators. To understand these, we need to examine if and how the other treatment groups were motivated, so we may compare with the Productivity group. To understand motivation, we evaluate how workers perceived the wage increases (via the compensation changes) as well as other facets of the treatment: transparency, communication, and wage alignment. Then we examine what behavior changes workers made in order to increase productivity. Through this examination of motivation, we can determine what barriers or blockages the Productivity treatment group faced.

#### Worker perception of wages

**Fig 3:**
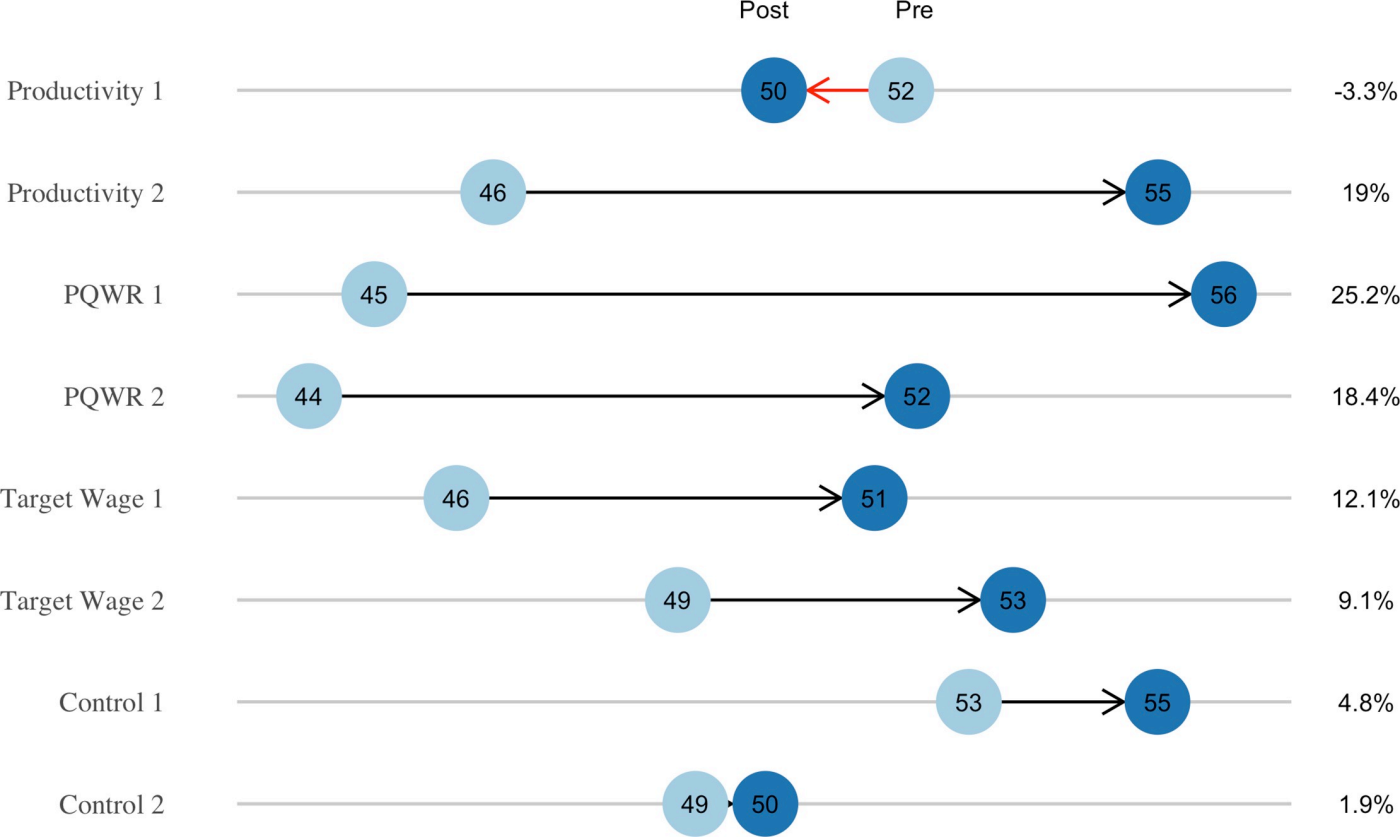
Change in average hourly wage between pre-treatment and post-treatment periods for each line. The right-hand side shows percentage change. “1” and “2” denote the line.

We assume that workers were not able to precisely parse changes in wages due to the treatment versus changes due to other causes, such as longer orders, more familiar styles, and so forth. The direct juxtaposition of new and old compensation on each biweekly paycheck increased the likelihood that workers attributed most wage increases to the treatment. This supposition is supported by evidence from the focus groups that workers did attribute their wage increases to the treatment. Fig 3 shows the total hourly wage change.

In the first focus group, many sewing line workers said it would be “fair” to be paid 500 baht in 8 hours, but that it would be “good” if they received 700 baht for the whole workday. We might suggest the “good” level is analogous to a living wage, though we did not try to precisely measure this figure. For comparison, the Asian Floor Wage was estimated at 570 baht in 2016 and 606 baht in 2017. The treatment did raise average wages above the “fair” level (albeit not within 8 hours), but not above the “good” level. As a result, we should expect continued dissatisfaction with wages even as the wage increases were highly welcomed. Indeed, most workers who received the treatment, even the Productivity group, spoke positively about their new wages, though that they would still like to earn more.

And yet, in the second focus group, a worker from Productivity line 1 did say: “I thought that I could have earned more than this". Workers on that line were apparently earning only half the incentive earned by Productivity line 2. Interestingly, in the third and fourth focus groups, this discrepancy did not resurface outwardly. This might result from workers growing accustomed to new wage potentials, and perhaps the effort required to overcome their particular barriers was not worth the potential gain in income, or perhaps they felt it was out of their control.

The case of the Target wage treatment is instructive in this regard, since the target wage could be compared conceptually with a reference wage. If the Target wage group reached their reference wage and were presented with the opportunity to go home, then we might expect them to elect to do so at least some of the time [37]. Indeed, before the intervention, workers reported a desire to work fewer days and shorter hours (although not at the expense of wages). Referencing OLS specification 6 in Table 3, the Target wage treatment shows a larger hourly wage increase (4.6 baht) than the PQWR (3.7 baht) and Productivity (2.1 baht) treatments. This increase, while larger, raised Target wage’s average wage to comparable levels with the other treatment groups, yet neither line in the Target wage group ever opted to go home early. Assuming reasonably homogenous wage preferences, the Target wage group’s decisions should be representative of other factory workers.

We might expect this outcome. The first target wage, 500 baht in eight hours, while “fair”, is significantly below what workers believe is “good” for a workday. The second target, 650 baht in ten hours, is perhaps more salient, but workers would want that wage *on average*. Because an equivalently satisfying wage is not guaranteed each day, workers seek to maximize earnings on days of high-earning potential, instead of taking off early. Income is such an issue that a number of workers held secondary jobs, such as running a small shop or selling items to other workers, on top of the 60-hour factory week. In addition, if the workers left early, they would have the extra costly task of rearranging rides, alerting family members, and coordinating plans. As it stood, workers were motivated by long days with higher earning potential, a sentiment noted in the focus groups, despite a desire to spend more time with family and friends. This relationship can be seen in the data by looking at the non-linear working hour effect represented by overtime: for both productivity and wages, overtime is positive and significant.

#### Worker perception of other treatment features

Before looking at changes in worker behavior, we need to understand the effect of the other treatment features. One major feature of the treatment was the communication and transparency measures. Overall, workers reported a similar level of understanding as with the pre-treatment compensation system: the incentive was difficult to calculate while working, but they understood the mechanics. Similarly, the updated LCD monitors were better than before, but still limited. For instance, the information—such as expected wage—was occasionally wrong, especially when the number of workers on the line fluctuated during the day. This frustrated workers and made them somewhat distrustful of the monitors. They preferred to consult a sheet posted on a board at the end of the line or to confer with fellow workers. Yet, later on, management added a red/green color-coding on the monitor to readily display if a target was met and how many garments it would require to meet the next target. Workers were encouraged by this easy-to-understand display. It is unlikely that these aspects of transparency provided much additional benefit, though they did not detract from the treatment either.

The communication procedures could also support another effect: simply being observed can affect changes in behavior. The Hawthorne Effect, as it is known, would require workers to know they are being observed, and perceive that observation as significant to their aims as to elicit a change in behavior [54]. The evidence here is mixed. First, the effect on productivity from the treatment is immediate and persists (see Tables J-M in S1 Appendix for short- and medium-term regression summaries). We might expect the Hawthorne Effect to diminish once the honeymoon period had ended, yet workers did consistently note in focus groups that they knew they were under observation. However, this feeling of being observed had multiple effects on their motivation. First, workers initially expressed distrust of management. They believed if they worked harder, management would raise the goals and reduce the incentive. Second, workers in later focus groups reported feeling increasingly positive feelings towards management. They felt like management cared, which made them want to work harder out of a sense of reciprocity. Third, workers acknowledged that their colleagues in the factory likely would be envious of the treatment, so they encouraged the factory to provide it to all workers as soon as possible.

Finally, the treatment aligned incentives for workers along the production chain—from cutters and mechanics, to sewers, quality inspectors, and team leaders. We see limited evidence that the incentive induced significant changes for several of these worker-groups. For instance, cutters were still dissatisfied with their wages. Further, their physical disconnection from the lines meant they were unaware about the line’s production (and thus their wage) throughout the day. The relief team, instead of being more motivated and/or supported, reported feeling extra pressure from the line they supported and would be blamed for production issues. Moreover, the line workers reported that there was still the same level of issues with materials and machines. This suggests that the workers responsible had not changed their behavior significantly. Overall, certain physical and workplace features limited the effectiveness of the incentive alignment.

#### Worker behavior changes

Workers are able to control their effort in the form of work-rate, time spent working, quality of work, and type of work, including supporting their team members. Workers can also stay at the job or quit. It appears that the increased wages and attention from management were motivating. However, it is not obvious which behavior changes led to the increase in productivity.

#### No evidence for a higher work-rate

We do not find evidence for significant change in work-rate. Workers across all groups did not report working any faster. Some, in focus groups, did say their colleagues appeared to be less “lazy” and “tried harder”, but none mentioned anything about speed. We might expect stress to increase with work-rate, but in focus groups, workers did not note any significant change in their stress levels, either positively or negatively due to the intervention.

#### Some evidence for more time spent working

If workers show up for work, do so on time, and take fewer breaks, their productivity, all else equal, should increase. We find some evidence that workers reduced their tardiness due to the treatment. Table N in S1 Appendix shows a significant decrease in tardiness for the PQWR treatment group due to the treatment. Workers reported that fellow workers were less tardy, and that their colleagues took fewer and/or shorter breaks (though we do not have data on breaks). Many workers did report that they were more likely to show up to work because of the potential to earn more. As one worker reported: “Team is… on time and willing to work overtime and help each other because they know that the more they work, the more they earn. And if they simply take leave, they might, sometimes, miss 700 baht in a day” (Focus group, 9/1/16). We do not find any evidence for changes in absenteeism. This may be due to the fact that day-to-day absences are likely driven by contingencies, such as a sick child or personal illness.

#### Substantial evidence for greater attention to quality

There is substantial evidence that workers increased their attention to detail. Quality issues can significantly hamper productivity, and are frustrating and aggravating for all workers. To reduce the number of defects, aside from skill enhancements, workers consult with quality inspectors to better understand quality standards and they can take care that each garment meets the quality standard. In the focus groups, workers noted they took both these actions. And in the data, we see that the quality rate increased significantly. For both the PQWR and Target wage treatments, we find significant, positive increases of about 1.8%-points in the quality rate, as shown in Table 4. The Productivity treatment, however, saw no effect.

**Table 4:** OLS regressions with quality rate as the dependent variable.

	*Dependent variable*:					
	Quality Rate (%)					
	1	2	3	4	5	6
**Productivity treatment**	0.797	0.808	0.8	0.745	0.853	0.867
	(0.515)	(0.519)	(0.541)	(0.549)	(0.562)	(0.561)
**PQWR treatment**	1.814***	1.820***	1.707***	1.653***	1.677***	1.663***
	(0.533)	(0.538)	(0.551)	(0.545)	(0.554)	(0.555)
**Target wage treatment**	1.816***	1.820***	1.716***	1.638***	1.771***	1.768***
	(0.451)	(0.451)	(0.480)	(0.487)	(0.481)	(0.479)
**Overtime**		0.125	0.127	0.125	0.294	0.287
		(0.275)	(0.276)	(0.280)	(0.454)	(0.455)
**Familiarity**			0.005**	0.005**	0.005**	0.004**
			(0.002)	(0.002)	(0.002)	(0.002)
**Number of line workers**				1.127	0.62	0.305
				(3.988)	(4.067)	(4.091)
**Square of Number of line workers**				-4.889**	-5.110**	-5.078**
				(2.399)	(2.473)	(2.465)
**Cube of Number of line workers**				-3.856	-4.209*	-4.218*
				(2.345)	(2.390)	(2.391)
**SAM**					-0.105*	-0.105*
					(0.054)	(0.054)
**Work hours**					-0.069	-0.065
					(0.126)	(0.127)
**Style—consecutive days in production**					0.019	0.019
					(0.015)	(0.015)
**Tardiness (mins)**						-0.003*
						(0.002)
**Total absenteeism (%)**						-0.003
						(0.004)
**Observations**	4,191	4,191	4,191	4,191	4,191	4,191

Note

*p<0.1

**p<0.05

***p<0.01

Standard errors corrected for serial correlation using Newey-West variance estimator.

Fixed effects for each specification are date, style, and line.

Similarly, when the quality rate is included as a covariate in the regressions on productivity, as seen in Table O in S1 Appendix, the treatment effect is greatly diminished. This suggests that changes in the quality rate account for a large portion of the behavior change induced by the treatment. This makes sense that workers would adjust their quality rate: it is largely under their control and provides a high productivity return on effort. Indeed, they are already producing a fairly high quality.

An alternative explanation is that because the quality inspector is now incented by productivity, the inspector might lower (nearly undetectably) quality standards. In the short term and with a weaker buyer-supplier relationship, this would be difficult to rule out. However, the buyer-supplier relationship is strong, so it is unlikely that poor quality garments would have gone unnoticed for a full year.

#### Minimal evidence for taking on additional responsibility

In addition to improving the quality of their work, workers may also take on extra responsibility, such as supporting team members, fixing machines, and so forth, to support productivity. We find minimal evidence that this took place. Line managers reported that workers displayed more problem-solving initiative. But in the larger picture, workers were still highly aggravated by the free rider issue endemic to group rates and teamwork. Nearly all workers advocated for some skill or seniority wage bonuses to account for these effects. While workers supported their fellow workers at times, there was still animosity that prevented more widespread collaboration.

Moreover, workers were constrained by hierarchical management and a lack of skills to perform other tasks that needed to be done. The other major enablers of productivity were related to line balancing, materials, and machines. The team leader controls line balancing, and while workers reportedly offered to help given they had intimate knowledge of their colleagues’ abilities, they were largely ignored. Similarly, workers were frustrated by inadequate material supply, and they offered numerous suggestions to alleviate problems, but did not affect change on a consistent basis. And finally, workers were still largely reliant on mechanics for major machine fixes, which caused major problems particularly when mechanics left during overtime.

#### Significant evidence for reduced turnover

We find significant evidence for reduced turnover. Many workers noted that the factory had a reputation for paying higher wages than comparable jobs, and that the wage intervention only bolstered the factory’s position. Turnover data for the two periods is shown in Table 5 below.

**Table 5:** Average monthly turnover per year per line.

Line	2015	2016
**Productivity 1**	6.21	6.7
**Productivity 2**	3.91	4.04
**PQWR 1**	1.93	1.98
**PQWR 2**	6.71	4.93
**Target Wage 1**	2.72	1.9
**Target Wage 2**	3.12	0.79
**Control 1**	1.14	1.72
**Control 2**	2.71	5.28

The turnover rate is the number of workers who left divided by the number of workers who were on the line at the beginning of the month. This does not track individual workers, so it may be the newest workers who are leaving each month. A rate just above 5% indicates that about one worker left per month. “1” and “2” denote the line.

Reduced turnover benefits productivity due to skill and experience benefits. Workers at the factory are more likely to be more skilled than those who might be hired. Retained workers can also build on their skills to become more multi-skilled; this generates large benefits for line-balancing. Retained workers also gain tacit knowledge about the factory, machines, colleagues, material, and so forth, that can benefit productivity.

#### Lessons

The workers appeared to know how to increase their productivity. They routinely made suggestions to support production flow, from adding workers, to helping materials delivery, to improving line balancing, to improving the mechanic response-time. Since line workers were not able to control those facets of production, they increased effort where it would yield the highest benefit: attention to quality and tardiness. Of course, the Productivity treatment group did not increase their quality, nor their productivity. As shown in Tables P-R of the S1 Appendix, the Productivity treatment group’s productivity significantly decreased when compared to the other treatment groups, while its wages neither increased nor decreased.

A major difference that may have caused this discrepancy between Productivity and the other treatment groups was line-level turnover. The Productivity lines had persistently higher turnover. We do not have individual-level data to examine, but we speculate that higher turnover lowered the average skill and experience level of the line, depressing wage potentials, and reducing the incentive for those workers to put in extra effort where they could: by increasing quality.

Overall, most workers were motivated by the potential for increased wages from an accelerating group rate as well as increased engagement and sense of fair compensation. Workers focused their increased effort on reducing quality defects and tardiness, two behaviors which individual workers largely control. Additional productivity-increasing behaviors were constrained by skill, position, and conflicts arising from free riders.

### Impact on profit

We next examine the compensation system effect on factory profit. The increased productivity does not guarantee increased profit, as workers receive a higher share of the per-garment value, on average. Because we lack pre-treatment data on prices, we estimated the profit-per-garment decrease through a cross-sectional OLS over the post-treatment period (see Table S in S1 Appendix). Though our estimates may suffer from omitted variable bias due to uncontrolled-for intangible differences between lines, we are reasonably confident in the results, because there is a fairly mechanistic story. Per-garment profits decreased by about three baht for each line.

We then constructed a simple profit assessment from mean changes in output and profit per-garment. While mostly illustrative, it demonstrates that productivity gains did overcome losses in per-garment profit, yielding a higher total profit. Table 6 shows that five out of the six treatment lines realized increases.

**Table 6:** A simple profit assessment.

	Productivity 1	Productivity 2	PQWR 1	PQWR 2	Target Wage 1	Target Wage 2	Control 1	Control 2
**Pre-Treatment Output per Day**	603	508	463	390	504	476	667	655
**Post-Treatment Output per Day**	446	602	535	474	571	579	543	563
**Pre-Treatment Profit per Garment**	29	48	54	54	52	62	61	39
**Post-Treatment Profit per Garment**	25	45	51	51	49	59	61	39
**Pre-Treatment Profit per Day**	17388	24375	25156	21232	26266	29655	40872	25517
**Post-Treatment Profit per Day**	11468	26504	26722	25063	27254	35638	33336	22846
**Profit per Day Change**	-5920	2129	1566	3831	988	5982	-7536	-2671

An estimate for the change in total profit between the two periods is derived using average output and OLS-derived profit-per-garment. “1” and “2” denote the line.

In addition, there are significant costs from employee turnover, management expenses, and capital investments. We rule out any effect of capital investments since any changes that happened were exogenously driven. And management expenses are relatively small compared to employee turnover costs, especially when spillover effects are considered. Employee turnover generates large costs for hiring and training, as well as lost productivity when a line has reduced numbers of workers. Given the decreases in turnover, the factory likely benefitted from the intervention. As evidence, the factory management proliferated the intervention across the factory’s 23 lines once the intervention ended.

## Summary

There remain significant tensions between workers, factory management, and brands regarding wages. Even as worker productivity has increased, the financial gains have not been shared with workers. When workers realize they are not receiving financial benefits from increased effort, problem solving, or skills development, they are likely to lose motivation and/or quit [55].

Skilled, engaged, and motivated workers are critical to respond to industry demands for agile production, mass customization, and even new forms of automation. This research provides evidence that paying workers more, delivered through a more transparent, fair, and Lean-aligned compensation system, can be a vehicle to raise worker satisfaction, reduce turnover, and increase productivity and profits.

This early work points to the need for additional research. A randomized control trial across more factories would yield greater insights for how new compensation systems respond under different conditions. Moreover, future research could examine worker responses to different levels of compensation and versions of compensation—including skill-based, seniority-based, and quality-based measures. Applying these lessons across factories and industries will require improved factory management, more sophisticated human resources data management, careful efforts to align factory goals with incentives, and improved communication and transparency.

This research suggests that, even with current factory margins and market conditions, important wage gains are possible and profitable. And yet, other efforts are still needed to progress to living wages. Brand sourcing practices—with respect to delivery times, quality requirements, and styles—need to be examined and improved to support worker wage increases [9,56]. Unions need to be supported to negotiate and protect wage gains, and to ratchet up working conditions over time [57,58]. Domestic regulators need to more consistently and strongly enforce existing labor law [59]. And as ILO’s Better Work program has demonstrated, governments should support economic and social upgrading with innovative policies, such as, in this case, more strategic compensation and upskilling programs [60,61].

## Supporting information

S1 Appendix Contains all supporting information, including tables and figures. **Table A. Summary statistics of Productivity Treatment Group. Table B. Summary statistics of PQWR Treatment Group. Table C. Summary statistics of Target Wage Treatment Group. Table D. Summary statistics of Control Group. Table E**. **Results from two-tailed t-test for Parallel Trends Assessment.** Comparison of simple time trend and time trend with controls for line, style, and number of workers. The first test shows a violation of the parallel trends assumption, however, the parallel trends assumption is not violated in the specification including controls.**Fig A**. **Time-Trend Plot for Parallel Trends Pre-Treatment Assessment.** Compare residuals of parallel trends regression with controls for line, style, and number of workers. No heterogeneity found in residuals. Target Wage residuals continue until mid-January 2016, because the Target Wage intervention did not start until that time. **Table F. Comparison by age (years) across lines in January 2016.** Line compositions changed over the course of the two-year period. **Table G. Comparison by length of employment (years) across lines in January 2016.** Line compositions changed over the course of the two-year period. **Table H. Comparison by sex across lines in January 2016.** Line compositions changed over the course of the two-year period. **Table I. Line manager characteristics in January 2016.** Line managers did not change throughout the two-year period. One line-manager managed both lines in each treatment group. **Table J**. **Shortest-term OLS regression with productivity as the dependent variable.** Short-term regression considers data until February 1^st^, 2016. Fixed effects for each specification are date, style, and line. **Table K. Second shortest-term OLS regression with productivity as the dependent variable.** Short-term regression considers data until March 1^st^, 2016. Fixed effects for each specification are date, style, and line. **Table L**. **Short-term OLS regression with productivity as the dependent variable.** Short-term regression considers data until May 1^st^, 2016. Fixed effects for each specification are date, style, and line. **Table M**. **Medium-term OLS regressions with productivity as the dependent variable.** Medium-term regression considers data until September 1^st^, 2016. Fixed effects for each specification are date, style, and line. **Table N**. **OLS regressions with tardiness as the dependent variable.** Fixed effects for each specification are date, style, and line. **Table O. OLS regressions including demeaned quality rate with productivity as the dependent variable.** Fixed effects for each specification are date, style, and line. The treatment effect disappears once quality is accounted for in the regression, which suggests that changes in the quality rate account for a large portion of the behavior change induced by the treatment. **Table P. Productivity-treatment-only OLS regressions with productivity as the dependent variable.** Fixed effects for each specification are date, style, and line. **Table Q. Productivity-treatment-only OLS regressions with quality rate as the dependent variable.** Fixed effects for each specification are date, style, and line. **Table R. Productivity-treatment-only OLS regressions with hourly wage as the dependent variable.** Fixed effects for each specification are date, style, and line. **Table S**. **OLS regressions on profit-per-garment.** This is not a Difference-in-Difference identification strategy, because the data only covers the post-treatment period, thus we only have 1,692 observations. We also note that line was dropped as a fixed effect because of collinearity. The treatment effect is a cross-sectional comparison between the treatment group and control group in the post-treatment period.
(DOCX)
