# Supplementary material for: Factory benefits to paying workers more: The critical role of compensation systems in apparel manufacturing
Source: PLoS One. 2020 Feb 5;15(2):e0227510. doi: 10.1371/journal.pone.0227510 (PMC7001908; doi:10.1371/journal.pone.0227510)
Supplement: S1 Appendix — Table A. Summary statistics of Productivity Treatment Group. Table B. Summary statistics of PQWR Treatment Group. Table C. Summary statistics of Target Wage Treatment Group. Table D. Summary statistics of Control Group. Table E. Results from two-tailed t-test for Parallel Trends Assessment. Comparison of simple time trend and time trend with controls for line, style, and number of workers. The first test shows a violation of the parallel trends assumption, however, the parallel trends assumption is not violated in the specification including controls.Fig A. Time-Trend Plot for Parallel Trends Pre-Treatment Assessment. Compare residuals of parallel trends regression with controls for line, style, and number of workers. No heterogeneity found in residuals. Target Wage residuals continue until mid-January 2016, because the Target Wage intervention did not start until that time. Table F. Comparison by age (years) across lines in January 2016. Line compositions changed over the course of the two-year period. Table G. Comparison by length of employment (years) across lines in January 2016. Line compositions changed over the course of the two-year period. Table H. Comparison by sex across lines in January 2016. Line compositions changed over the course of the two-year period. Table I. Line manager characteristics in January 2016. Line managers did not change throughout the two-year period. One line-manager managed both lines in each treatment group. Table J. Shortest-term OLS regression with productivity as the dependent variable. Short-term regression considers data until February 1st, 2016. Fixed effects for each specification are date, style, and line. Table K. Second shortest-term OLS regression with productivity as the dependent variable. Short-term regression considers data until March 1st, 2016. Fixed effects for each specification are date, style, and line. Table L. Short-term OLS regression with productivity as the dependent variable. Short-term regression conside [file pone.0227510.s001.docx]

# **Supporting information**

**Data Analysis**

There are several covariates for which we do not have data that may be important to precisely estimate treatment effects. The most problematic omission is downtime, the length of time a line has stopped producing during normal working hours. The dataset contains downtime data based on the type—machine, material, personnel, or quality—for the pre-treatment period, but we were unable to acquire it for the post-treatment period. We ran multiple exploratory regressions over the pre-treatment period in order to understand how downtime would likely impact wages and productivity. We found no significant relationship between wage and any form of downtime. With productivity, however, total downtime was significant at the 10% level with an estimate of -0.025. The number of occurrences where this would affect productivity more than 1%-point was 708 and more than 5%-points was 33 instances of 2020 total observations. When explored by type of downtime, material-induced downtime (e.g., the line did not have fabric to sew) was significant at the 1% level with an estimate of -0.17. The number of instances where it reduced productivity more than 1%-point was 192 and more than 5%-points was 65 times out of 2020 total observations. The other types of downtime were not significant nor particularly large estimates.

In addition, the data contained a number of outliers, potentially the result of measurement error. Some values were obviously faulty, such as a value of unplanned absenteeism over 1000%. These instances were excluded from the dataset. We also flagged and removed instances which were highly implausible, such as where productivity was very high (greater than 150%) while the incentive was very low (less than 50 baht), or when productivity was very low (less than 55%) while the incentive was non-zero.

Other filtering decisions involved judgement and sensitivity analysis. For instance, we excluded observations where wages were recorded as less than minimum wage, number of workers was equal to zero or greater than 34, and productivity equal to zero or greater than 4 standard deviations from the mean (leaving a maximum of 178%). The overall statistical story was not affected when we ran the specified regressions with productivity maxima of 140%, 160%, 180%, and 200%, or three standard deviations and five standard deviations. In addition, we found no abnormal or asymmetric deviations when comparing the reported productivity with a productivity calculated using the SAM, output, number of workers, and other data.

**S1A Table. Summary statistics of Productivity Treatment Group.**

| Statistic | N | Mean | St. Dev. | Min | Pctl(25) | Pctl(75) | Max |
| --- | --- | --- | --- | --- | --- | --- | --- |
| Productivity (%) | 1,100 | 96.98 | 35.87 | 2 | 70 | 123 | 177 |
| Hourly Wage (THB) | 1,097 | 50.94 | 11.18 | 37.5 | 42.61 | 58.12 | 93.32 |
| Working Hours | 1,097 | 9.92 | 1.48 | 8 | 8 | 11 | 12 |
| Overtime (0,1) | 1,100 | 0.49 | 0.5 | 0 | 0 | 1 | 1 |
| Number of Workers | 1,100 | 19.34 | 1.75 | 11 | 18 | 20 | 28 |
| SAM (min/garment) | 1,099 | 21.99 | 8.07 | 1.84 | 17.11 | 26.35 | 56.92 |
| Lead Time (days) | 1,096 | 6.56 | 7.92 | 1 | 2 | 8 | 57 |
| Style Familiarity (style-days/line) | 1,100 | 59.07 | 57.75 | 1 | 16 | 75 | 179 |
| Tardiness (min) | 1,094 | 5.87 | 25.57 | 0 | 0 | 5 | 725 |
| Unplanned Absenteeism (%) | 1,100 | 2.16 | 3.55 | 0 | 0 | 4.5 | 30 |

**S1B Table. Summary statistics of PQWR Treatment Group.**

| Statistic | N | Mean | St. Dev. | Min | Pctl(25) | Pctl(75) | Max |
| --- | --- | --- | --- | --- | --- | --- | --- |
| Productivity (%) | 1,084 | 93.35 | 35.01 | 2 | 68 | 118 | 178 |
| Hourly Wage (THB) | 1,084 | 49.31 | 11.17 | 37.5 | 42.61 | 55.06 | 93.35 |
| Working Hours | 1,084 | 10.03 | 1.47 | 8 | 8 | 11 | 12 |
| Overtime (0,1) | 1,084 | 0.53 | 0.5 | 0 | 0 | 1 | 1 |
| Number of Workers | 1,084 | 19.2 | 1.77 | 10 | 18 | 21 | 28 |
| SAM (min/garment) | 1,084 | 24.11 | 4.2 | 1.84 | 21.51 | 26.35 | 41.25 |
| Lead Time (days) | 1,082 | 8.06 | 8.15 | 1 | 2 | 12 | 43 |
| Style Familiarity (style-days/line) | 1,084 | 54.51 | 40.93 | 1 | 17 | 99 | 124 |
| Tardiness (min) | 1,083 | 4.34 | 13.75 | 0 | 0 | 4 | 302 |
| Unplanned Absenteeism (%) | 1,084 | 1.8 | 3.41 | 0 | 0 | 4.51 | 39.47 |

**S1C Table.** **Summary statistics of Target Wage Treatment Group.**

| Statistic | N | Mean | St. Dev. | Min | Pctl(25) | Pctl(75) | Max |
| --- | --- | --- | --- | --- | --- | --- | --- |
| Productivity (%) | 1,147 | 95.46 | 35.26 | 2 | 72 | 120 | 178 |
| Hourly Wage (THB) | 1,146 | 49.85 | 11.3 | 37.5 | 42.61 | 55.37 | 111.49 |
| Working Hours | 1,146 | 9.95 | 1.44 | 8 | 8 | 11 | 12 |
| Overtime (0,1) | 1,147 | 0.52 | 0.5 | 0 | 0 | 1 | 1 |
| Number of Workers | 1,147 | 20.44 | 1.4 | 14 | 20 | 21 | 27 |
| SAM (min/garment) | 1,147 | 21.83 | 3.86 | 1.62 | 20.66 | 23.02 | 38.03 |
| Lead Time (days) | 1,143 | 6.11 | 6.42 | 1 | 2 | 8 | 34 |
| Style Familiarity (style-days/line) | 1,147 | 43.42 | 39.91 | 1 | 17 | 50 | 141 |
| Tardiness (min) | 1,147 | 4.17 | 12.8 | 0 | 0 | 4 | 247 |
| Unplanned Absenteeism (%) | 1,147 | 1.34 | 3.61 | 0 | 0 | 0 | 77 |

**S1D Table.** **Summary statistics of Control Group.**

| Statistic | N | Mean | St. Dev. | Min | Pctl(25) | Pctl(75) | Max |
| --- | --- | --- | --- | --- | --- | --- | --- |
| Productivity (%) | 1,099 | 106.5 | 9 32.69 | 3 | 86 | 128 | 174 |
| Hourly Wage (THB) | 1,098 | 51.8 | 9.69 | 37.5 | 0 42.61 | 58.12 | 99.1 |
| Working Hours | 1,098 | 9.97 | 1.46 | 8 | 8 | 11 | 12 |
| Overtime (0,1) | 1,099 | 0.53 | 0.5 | 0 | 0 | 1 | 1 |
| Number of Workers | 1,099 | 21.16 | 1.93 | 9 | 20 | 22 | 28 |
| SAM (min/garment) | 1,080 | 22.87 | 7.62 | 0 | 18.91 | 26.35 | 78 |
| Lead Time (days) | 1,083 | 5.94 | 6.13 | 1 | 2 | 8 | 34 |
| Style Familiarity (style-days/line) | 1,099 | 59.83 | 49.42 | 1 | 16 | 96 | 154 |
| Tardiness (min) | 1,099 | 5.39 | 23.38 | 0 | 0 | 3 | 274 |
| Unplanned Absenteeism (%) | 1,099 | 1.74 | 4.02 | 0 | 0 | 2.3 | 75 |

**S1E Table**. **Results from two-tailed t-test for Parallel Trends Assessment.**

| Dependent Variable | Treatment Group | Simple Time Trend P-value | Time Trend w/ Controls P-value |
| --- | --- | --- | --- |
| Productivity (%) | Productivity | 0 | 0.62 |
|  | PQWR | 0 | 0.11 |
|  | Target Wage | 0 | 0.31 |
| Hourly Wage (THB) | Productivity | 0 | 0.6 |
|  | PQWR | 0 | 0.12 |
|  | Target Wage | 0 | 0.14 |

**S1A Fig**. **Time-Trend Plot for Parallel Trends Pre-Treatment Assessment.**


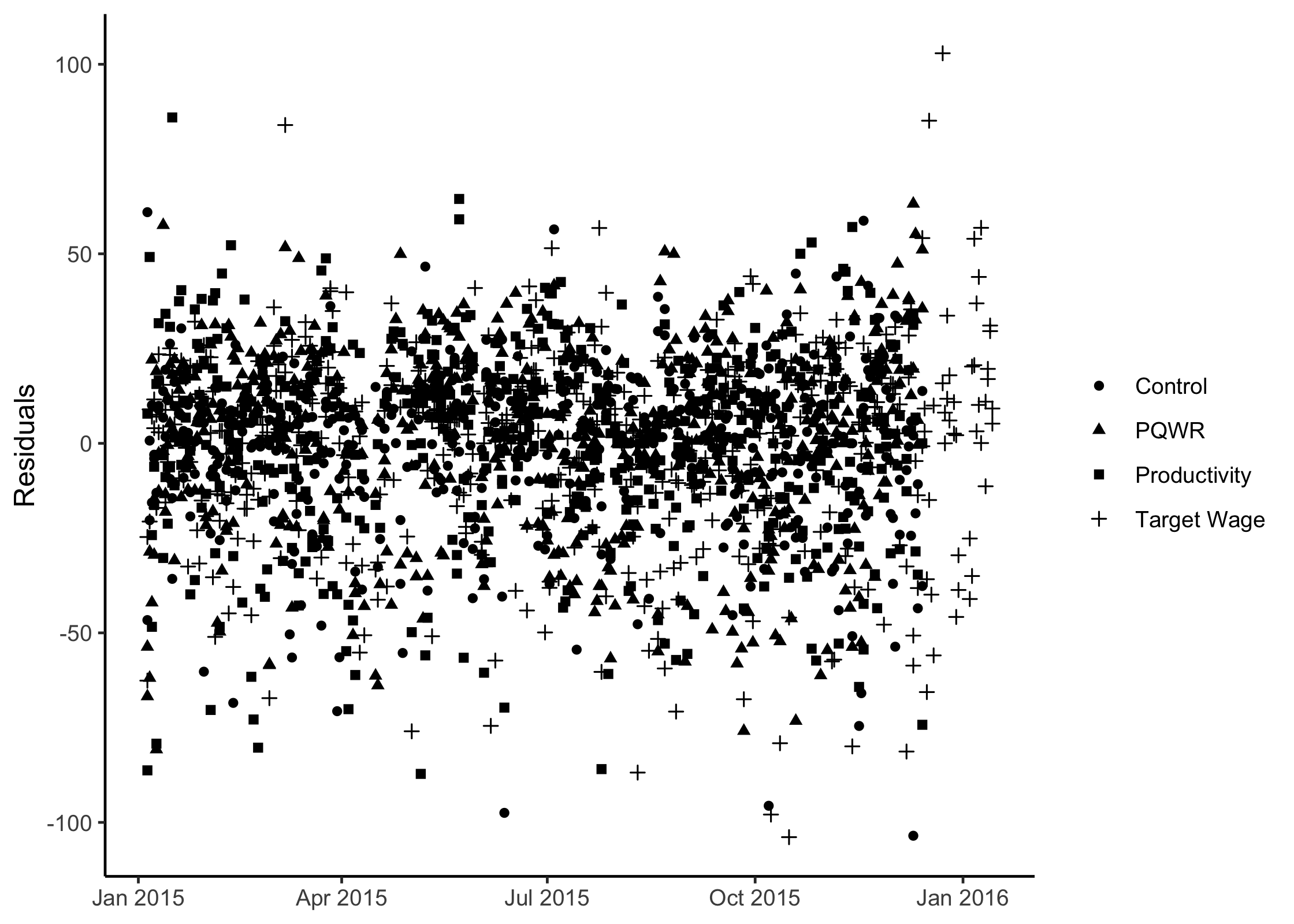


**S1F Table.** **Comparison by age (years) across lines in January 2016.**

| Statistic | N | Mean | St. Dev. | Min | Max |
| --- | --- | --- | --- | --- | --- |
| Productivity 1 | 20 | 36 | 6.7 | 23 | 52 |
| Productivity 2 | 17 | 35.5 | 8.5 | 19 | 44 |
| PQWR 1 | 19 | 34.8 | 8.1 | 24 | 49 |
| PQWR 2 | 19 | 35.7 | 5.9 | 24 | 46 |
| Target Wage 1 | 20 | 38 | 7.3 | 24 | 52 |
| Target Wage 2 | 21 | 39.5 | 8 | 23 | 52 |
| Control 1 | 21 | 34.8 | 7.7 | 21 | 52 |
| Control 2 | 19 | 36.7 | 8.8 | 23 | 52 |

**S1G Table.** **Comparison by length of employment (years) across lines in January 2016.**

| Statistic | N | Mean | St. Dev. | Min | Max |
| --- | --- | --- | --- | --- | --- |
| Productivity 1 | 19 | 6.41 | 6.22 | 0.01 | 18.03 |
| Productivity 2 | 20 | 2.92 | 2.77 | 0.07 | 9.11 |
| PQWR 1 | 21 | 7.42 | 4.67 | 0.03 | 18 |
| PQWR 2 | 21 | 5.67 | 5.7 | 0.03 | 18.02 |
| Target Wage 1 | 17 | 3.81 | 4.73 | 0.01 | 14.04 |
| Target Wage 2 | 18 | 6.27 | 5.65 | 0.01 | 14.03 |
| Control 1 | 19 | 4.36 | 4.26 | 0.01 | 12.02 |
| Control 2 | 20 | 5.44 | 4.21 | 0.04 | 18 |

**S1H Table.** **Comparison by sex across lines in January 2016.**

| Line | Men | Women |
| --- | --- | --- |
| Productivity 1 | 0 | 19 |
| Productivity 2 | 0 | 19 |
| PQWR 1 | 6 | 14 |
| PQWR 2 | 1 | 16 |
| Target Wage 1 | 0 | 20 |
| Target Wage 2 | 1 | 20 |
| Control 1 | 4 | 17 |
| Control 2 | 0 | 19 |

**S1I Table.** **Line manager characteristics in January 2016.**

| Treatment Group | Years at Facility | Years as Manager | No. of Trainings |
| --- | --- | --- | --- |
| Productivity | 19 | 19 | 6 |
| PQWR | 13 | 2 | 6 |
| Target Wage | 16 | 11 | 7 |
| Control | 18 | 10 | 6 |

**S1J Table**. **Shortest-term OLS regression with productivity as the dependent variable.**

|  | *Dependent variable:* | |  |  |  |  |
| --- | --- | --- | --- | --- | --- | --- |
|  | Productivity (%) | |  |  |  |  |
|  | 1 | 2 | 3 | 4 | 5 | 6 |
| Productivity treatment | 6.49 | 6.423 | 6.851 | 6.453 | 6.021 | 6.307 |
|  | (5.506) | (5.511) | (5.053) | (5.133) | (5.211) | (5.252) |
| PQWR treatment | 12.846^*^ | 12.913^**^ | 11.675^*^ | 12.028^*^ | 11.957^*^ | 11.966^*^ |
|  | (6.624) | (6.588) | (6.387) | (6.394) | (6.378) | (6.811) |
| Target wage treatment | 25.276^***^ | 25.239^***^ | 20.000^**^ | 19.824^**^ | 19.661^**^ | 19.450^**^ |
|  | (8.849) | (8.879) | (8.272) | (8.659) | (8.743) | (8.424) |
| Overtime |  | 2.57 | 3.007 | 3.224 | 3.232 | -5.552 |
|  |  | (2.383) | (2.231) | (2.270) | (2.277) | (7.986) |
| Familiarity |  |  | 0.178^***^ | 0.177^***^ | 0.176^***^ | 0.167^***^ |
|  |  |  | (0.029) | (0.030) | (0.030) | (0.030) |
| Number of line workers | |  |  | 22.028 | 20.494 | 22.414 |
|  |  |  |  | (26.569) | (26.544) | (25.594) |
| Square of Number of line workers | | |  | -15.328 | -16.998 | -19.778 |
|  |  |  |  | (33.418) | (33.418) | (32.560) |
| Cube of Number of line workers | | |  | -61.194^**^ | -60.880^**^ | -53.447^*^ |
|  |  |  |  | (30.192) | (30.107) | (28.206) |
| SAM |  |  |  |  | 0.847 | 0.864 |
|  |  |  |  |  | (0.622) | (0.648) |
| Work hours |  |  |  |  |  | 3.019 |
|  |  |  |  |  |  | (2.638) |
| Style - consecutive days in production | | |  |  |  | 0.241 |
|  |  |  |  |  |  | (0.153) |
| Tardiness (minutes) | |  |  |  |  | 0.048 |
|  |  |  |  |  |  | (0.041) |
| Total absenteeism (%) | |  |  |  |  | 0.035 |
|  |  |  |  |  |  | (0.121) |
| Observations | 2,237 | 2,237 | 2,237 | 2,237 | 2,237 | 2,237 |
| *Note:* | ^*^p<0.1; ^**^p<0.05; ^***^p<0.01 | | |  |  |  |
|  | Standard errors corrected for serial correlation using Newey-West variance estimator. | | | | | |

**S1K Table**. **Second shortest-term OLS regression with productivity as the dependent variable.**

|  | *Dependent variable:* | |  |  |  |  |
| --- | --- | --- | --- | --- | --- | --- |
|  | Productivity (%) | |  |  |  |  |
|  | 1 | 2 | 3 | 4 | 5 | 6 |
| Productivity treatment | 0.081 | 0.079 | -1.403 | -2.095 | -1.57 | -0.996 |
|  | (5.727) | (5.729) | (6.165) | (6.253) | (5.760) | (5.794) |
| PQWR treatment | 19.013^***^ | 19.060^***^ | 15.026^***^ | 15.192^***^ | 15.265^***^ | 15.759^***^ |
|  | (6.339) | (6.331) | (5.701) | (5.713) | (5.649) | (5.819) |
| Target wage treatment | 22.912^***^ | 22.917^***^ | 14.040^***^ | 13.819^***^ | 14.279^***^ | 15.906^***^ |
|  | (5.850) | (5.839) | (5.042) | (5.055) | (4.986) | (4.950) |
| Overtime |  | 2.746 | 3.255 | 3.463 | 3.472 | -4.884 |
|  |  | (2.437) | (2.274) | (2.322) | (2.327) | (6.736) |
| Familiarity |  |  | 0.172^***^ | 0.171^***^ | 0.170^***^ | 0.161^***^ |
|  |  |  | (0.027) | (0.027) | (0.027) | (0.028) |
| Number of line workers | |  |  | 15.8 | 14.876 | 18.022 |
|  |  |  |  | (27.933) | (27.657) | (27.319) |
| Square of Number of line workers | | |  | -15.341 | -17.304 | -18.246 |
|  |  |  |  | (34.921) | (34.959) | (34.933) |
| Cube of Number of line workers | | |  | -65.561^**^ | -65.515^**^ | -61.962^**^ |
|  |  |  |  | (28.963) | (28.922) | (28.758) |
| SAM |  |  |  |  | 1.156 | 1.179 |
|  |  |  |  |  | (0.735) | (0.766) |
| Work hours |  |  |  |  |  | 2.877 |
|  |  |  |  |  |  | (2.152) |
| Style - consecutive days in production | | |  |  |  | 0.270^*^ |
|  |  |  |  |  |  | (0.153) |
| Tardiness (minutes) | |  |  |  |  | 0.002 |
|  |  |  |  |  |  | (0.017) |
| Total absenteeism (%) | |  |  |  |  | 0.064 |
|  |  |  |  |  |  | (0.114) |
| Observations | 2,414 | 2,414 | 2,414 | 2,414 | 2,414 | 2,414 |
| *Note:* | ^*^p<0.1; ^**^p<0.05; ^***^p<0.01 | | |  |  |  |
|  | Standard errors corrected for serial correlation using Newey-West variance estimator. | | | | | |

**S1L Table**. **Short-term OLS regression with productivity as the dependent variable.**

|  | *Dependent variable:* | |  |  |  |  |
| --- | --- | --- | --- | --- | --- | --- |
|  | Productivity (%) | |  |  |  |  |
|  | 1 | 2 | 3 | 4 | 5 | 6 |
| Productivity treatment | -5.777 | -5.767 | -6.676 | -7.159 | -7.226 | -5.907 |
|  | (4.926) | (4.926) | (4.594) | (4.728) | (4.644) | (4.723) |
| PQWR treatment | 14.662^**^ | 14.677^**^ | 9.771^*^ | 9.739^*^ | 9.827^*^ | 10.251^*^ |
|  | (6.437) | (6.434) | (5.649) | (5.695) | (5.698) | (5.879) |
| Target wage treatment | 12.891^**^ | 12.897^**^ | 5.648 | 5.415 | 5.686 | 8.009 |
|  | (6.323) | (6.316) | (5.239) | (5.244) | (5.331) | (5.517) |
| Overtime |  | 1.852 | 2.352 | 2.41 | 2.414 | -1.436 |
|  |  | (2.358) | (2.190) | (2.213) | (2.236) | (5.370) |
| Familiarity |  |  | 0.178^***^ | 0.178^***^ | 0.174^***^ | 0.162^***^ |
|  |  |  | (0.026) | (0.026) | (0.026) | (0.027) |
| Number of line workers | |  |  | 24.227 | 22.118 | 21.092 |
|  |  |  |  | (30.013) | (29.632) | (29.513) |
| Square of Number of line workers | | |  | -25.34 | -28.812 | -29.31 |
|  |  |  |  | (33.544) | (33.328) | (32.704) |
| Cube of Number of line workers | | |  | -20.844 | -21.338 | -20.452 |
|  |  |  |  | (34.924) | (34.568) | (33.854) |
| SAM |  |  |  |  | 1.388^**^ | 1.417^*^ |
|  |  |  |  |  | (0.698) | (0.728) |
| Work hours |  |  |  |  |  | 1.256 |
|  |  |  |  |  |  | (1.662) |
| Style - consecutive days in production | | |  |  |  | 0.352^**^ |
|  |  |  |  |  |  | (0.147) |
| Tardiness (minutes) | |  |  |  |  | 0.007 |
|  |  |  |  |  |  | (0.023) |
| Unplanned absenteeism (%) | | |  |  |  | -0.078 |
|  |  |  |  |  |  | (0.160) |
| Observations | 2,932 | 2,932 | 2,932 | 2,932 | 2,932 | 2,932 |
| *Note:* | ^*^p<0.1; ^**^p<0.05; ^***^p<0.01 | | |  |  |  |
|  | Standard errors corrected for serial correlation using Newey-West variance estimator. | | | | | |

**S1M Table**. **Medium-term OLS regressions with productivity as the dependent variable.**

|  | *Dependent variable:* | |  |  |  |  |
| --- | --- | --- | --- | --- | --- | --- |
|  | Productivity (%) | |  |  |  |  |
|  | 1 | 2 | 3 | 4 | 5 | 6 |
| Productivity treatment | -1.017 | -0.686 | -2.831 | -4.397 | -5.246 | -5.089 |
|  | (4.020) | (4.075) | (3.507) | (3.597) | (3.648) | (3.670) |
| PQWR treatment | 13.608^***^ | 13.781^***^ | 9.946^**^ | 9.617^**^ | 9.260^*^ | 9.227^*^ |
|  | (4.854) | (4.850) | (4.617) | (4.722) | (4.725) | (4.782) |
| Target wage treatment | 20.407^***^ | 20.422^***^ | 16.338^***^ | 15.495^***^ | 14.810^**^ | 16.143^***^ |
|  | (5.823) | (5.825) | (6.071) | (5.933) | (5.900) | (5.925) |
| Overtime |  | 5.583^**^ | 5.653^***^ | 5.761^***^ | 5.811^***^ | 8.209^*^ |
|  |  | (2.252) | (2.134) | (2.160) | (2.173) | (4.329) |
| Familiarity |  |  | 0.133^***^ | 0.134^***^ | 0.132^***^ | 0.123^***^ |
|  |  |  | (0.028) | (0.028) | (0.028) | (0.029) |
| Number of line workers | |  |  | 61.484 | 67.001^*^ | 66.771^*^ |
|  |  |  |  | (40.769) | (40.355) | (40.363) |
| Square of Number of line workers | | |  | -38.123 | -32.994 | -30.425 |
|  |  |  |  | (34.881) | (34.461) | (34.149) |
| Cube of Number of line workers | | |  | -58.875^*^ | -50.439 | -50.294 |
|  |  |  |  | (31.656) | (31.541) | (31.274) |
| SAM |  |  |  |  | 1.311^**^ | 1.315^**^ |
|  |  |  |  |  | (0.600) | (0.615) |
| Work hours |  |  |  |  |  | -0.923 |
|  |  |  |  |  |  | (1.324) |
| Style - consecutive days in production | | |  |  |  | 0.259^**^ |
|  |  |  |  |  |  | (0.118) |
| Tardiness (minutes) | |  |  |  |  | 0.006 |
|  |  |  |  |  |  | (0.022) |
| Unplanned absenteeism (%) | | |  |  |  | -0.027 |
|  |  |  |  |  |  | (0.159) |
| Observations | 3,544 | 3,544 | 3,544 | 3,544 | 3,544 | 3,544 |
| *Note:* | ^*^p<0.1; ^**^p<0.05; ^***^p<0.01 | | |  |  |  |
|  | Standard errors corrected for serial correlation using Newey-West variance estimator. | | | | | |

**S1N Table**. **OLS regressions with tardiness as the dependent variable.**

|  | *Dependent variable:* | |  |  |  |  |
| --- | --- | --- | --- | --- | --- | --- |
|  | Tardiness (minutes) | |  |  |  |  |
|  | 1 | 2 | 3 | 4 | 5 | 6 |
| Productivity treatment | 2.617 | 2.757 | 2.8 | 3.034 | 3.09 | 2.939 |
|  | 3.037 | 3.044 | 2.967 | 3.033 | 3.057 | 3.024 |
| PQWR treatment | -5.213^**^ | -5.145^**^ | -4.578^**^ | -4.734^**^ | -4.720^**^ | -4.714^**^ |
|  | 2.253 | 2.246 | 2.088 | 2.071 | 2.074 | 2.076 |
| Target wage treatment | -3.237 | -3.19 | -2.669 | -2.546 | -2.517 | -2.756 |
|  | 2.103 | 2.1 | 1.929 | 1.916 | 1.918 | 1.941 |
| Overtime |  | 1.467^*^ | 1.456^*^ | 1.399 | 1.395 | -2.04 |
|  |  | 0.867 | 0.868 | 0.871 | 0.87 | 1.373 |
| Familiarity |  |  | -0.025^*^ | -0.025^*^ | -0.024^*^ | -0.024^*^ |
|  |  |  | 0.013 | 0.013 | 0.013 | 0.013 |
| Number of line workers | |  |  | -37.442^*^ | -37.706^*^ | -33.039 |
|  |  |  |  | 22.575 | 22.59 | 23.064 |
| Square of Number of line workers | | |  | 1.803 | 1.573 | 0.482 |
|  |  |  |  | 26.011 | 25.995 | 26.069 |
| Cube of Number of line workers | | |  | -15.099 | -15.406 | -16.226 |
|  |  |  |  | 25.35 | 25.339 | 25.022 |
| SAM |  |  |  |  | -0.063 | -0.06 |
|  |  |  |  |  | 0.063 | 0.063 |
| Work hours |  |  |  |  |  | 1.313^**^ |
|  |  |  |  |  |  | 0.526 |
| Style - consecutive days in production | | |  |  |  | -0.027 |
|  |  |  |  |  |  | 0.03 |
| Total absenteeism (%) | |  |  |  |  | 0.080^***^ |
|  |  |  |  |  |  | 0.022 |
| Observations | 4,191 | 4,191 | 4,191 | 4,191 | 4,191 | 4,191 |
| *Note:* | ^*^p<0.1; ^**^p<0.05; ^***^p<0.01 | | |  |  |  |
|  | Standard errors corrected for serial correlation using Newey-West variance estimator. | | | | | |

**S1O Table. OLS regressions including demeaned quality rate with productivity as the dependent variable.**

|  | *Dependent variable:* | |  |  |  |  |
| --- | --- | --- | --- | --- | --- | --- |
|  | Productivity (%) | |  |  |  |  |
|  | 1 | 2 | 3 | 4 | 5 | 6 |
| Productivity treatment | -2.004 | -1.687 | -1.905 | -3.326 | -5.391 | -5.31 |
|  | (3.902) | (3.943) | (3.330) | (3.217) | (3.371) | (3.376) |
| PQWR treatment | 12.393^**^ | 12.549^**^ | 9.697^*^ | 6.497 | 5.567 | 5.585 |
|  | (5.304) | (5.312) | (5.023) | (4.724) | (4.715) | (4.721) |
| Target wage treatment | 13.695^***^ | 13.801^***^ | 11.177^**^ | 8.012^*^ | 6.2 | 6.642 |
|  | (4.701) | (4.711) | (4.724) | (4.566) | (4.429) | (4.423) |
| Overtime |  | 3.327^*^ | 3.380^**^ | 3.171^**^ | 3.266^**^ | 3.559 |
|  |  | (1.746) | (1.707) | (1.569) | (1.564) | (3.304) |
| Familiarity |  |  | 0.124^***^ | 0.114^***^ | 0.114^***^ | 0.111^***^ |
|  |  |  | (0.024) | (0.023) | (0.023) | (0.023) |
| Quality Rate | |  |  | 1.855^***^ | 1.881^***^ | 1.876^***^ |
|  |  |  |  | (0.318) | (0.326) | (0.325) |
| Tardiness (minutes) | |  |  | -0.008 | -0.008 | -0.008 |
|  |  |  |  | (0.020) | (0.019) | (0.019) |
| Total absenteeism (%) | |  |  | -0.045 | -0.032 | -0.033 |
|  |  |  |  | (0.034) | (0.031) | (0.031) |
| Number of line workers | |  |  |  | 25.922 | 25.486 |
|  |  |  |  |  | (31.855) | (31.686) |
| Square of Number of line workers | | |  |  | -32.185 | -31.401 |
|  |  |  |  |  | (29.789) | (29.730) |
| Cube of Number of line workers | | |  |  | -66.837^***^ | -65.963^***^ |
|  |  |  |  |  | (24.207) | (23.944) |
| SAM |  |  |  |  | 1.419^***^ | 1.420^***^ |
|  |  |  |  |  | (0.445) | (0.450) |
| Work hours |  |  |  |  |  | -0.124 |
|  |  |  |  |  |  | (1.086) |
| Style - consecutive days in production | | |  |  |  | 0.097 |
|  |  |  |  |  |  | (0.110) |
| Observations | 4,191 | 4,191 | 4,191 | 4,191 | 4,191 | 4,191 |
| *Note:* | ^*^p<0.1; ^**^p<0.05; ^***^p<0.01 | | |  |  |  |
|  | Standard errors corrected for serial correlation using Newey-West variance estimator. | | | | | |

**S1P Table. Productivity-treatment-only OLS regressions with productivity as the dependent variable.**

|  | *Dependent variable:* | |  |  |  |  |
| --- | --- | --- | --- | --- | --- | --- |
|  | Productivity (%) | |  |  |  |  |
|  | 1 | 2 | 3 | 4 | 5 | 6 |
| Productivity treatment | -9.117^**^ | -8.889^**^ | -7.519^**^ | -7.704^**^ | -8.696^***^ | -8.657^***^ |
|  | (3.630) | (3.668) | (3.052) | (3.063) | (3.109) | (3.113) |
| Overtime |  | 3.062^*^ | 3.180^*^ | 2.823^*^ | 2.923^*^ | 3.472 |
|  |  | (1.746) | (1.698) | (1.568) | (1.568) | (3.297) |
| Familiarity |  |  | 0.134^***^ | 0.125^***^ | 0.122^***^ | 0.120^***^ |
|  |  |  | (0.024) | (0.023) | (0.023) | (0.023) |
| Quality Rate | |  |  | 1.868^***^ | 1.903^***^ | 1.899^***^ |
|  |  |  |  | (0.324) | (0.333) | (0.332) |
| Turnover |  |  |  | 57.486 | 51.537 | 50.18 |
|  |  |  |  | (37.205) | (36.917) | (37.095) |
| Square Turnover | |  |  | -41.297 | -35.688 | -34.99 |
|  |  |  |  | (31.123) | (31.258) | (31.534) |
| Cube Turnover | |  |  | -34.403 | -35.05 | -34.763 |
|  |  |  |  | (28.332) | (28.447) | (28.525) |
| Number of line workers | |  |  | 27.861 | 32.924 | 30.677 |
|  |  |  |  | (33.009) | (32.525) | (32.516) |
| Square of Number of line workers | | |  | -41.599 | -35.93 | -35.216 |
|  |  |  |  | (29.190) | (29.299) | (29.309) |
| Cube of Number of line workers | | |  | -81.934^***^ | -74.081^***^ | -73.528^***^ |
|  |  |  |  | (22.319) | (23.001) | (22.802) |
| SAM |  |  |  |  | 1.413^***^ | 1.414^***^ |
|  |  |  |  |  | (0.449) | (0.452) |
| Work hours |  |  |  |  |  | -0.21 |
|  |  |  |  |  |  | (1.074) |
| Style - consecutive days in production | | |  |  |  | 0.063 |
|  |  |  |  |  |  | (0.111) |
| Tardiness (minutes) | |  |  |  |  | -0.009 |
|  |  |  |  |  |  | (0.019) |
| Total absenteeism (%) | |  |  |  |  | -0.031 |
|  |  |  |  |  |  | (0.031) |
| Observations | 4,191 | 4,191 | 4,191 | 4,191 | 4,191 | 4,191 |
| *Note:* | ^*^p<0.1; ^**^p<0.05; ^***^p<0.01 | | |  |  |  |
|  | Standard errors corrected for serial correlation using Newey-West variance estimator. | | | | | |

**S1Q Table. Productivity-treatment-only OLS regressions with quality rate as the dependent variable.**

|  | *Dependent variable:* | |  |  |  |  |
| --- | --- | --- | --- | --- | --- | --- |
|  | Quality Rate (%) | |  |  |  |  |
|  | 1 | 2 | 3 | 4 | 5 | 6 |
| Productivity treatment | -0.189 | -0.182 | -0.115 | -0.14 | -0.079 | 0.243 |
|  | (0.425) | (0.427) | (0.431) | (0.443) | (0.449) | (0.434) |
| Overtime |  | 0.087 | 0.093 | 0.086 | 0.269 | 0.125 |
|  |  | (0.277) | (0.277) | (0.279) | (0.452) | (0.419) |
| Familiarity |  |  | 0.007^***^ | 0.007^***^ | 0.006^***^ | 0.002 |
|  |  |  | (0.002) | (0.002) | (0.002) | (0.002) |
| Turnover |  |  |  | 0.14 | 0.291 | -1.487 |
|  |  |  |  | (5.851) | (5.873) | (5.120) |
| Square Turnover | |  |  | -1.706 | -1.853 | -0.566 |
|  |  |  |  | (4.694) | (4.770) | (4.152) |
| Cube Turnover | |  |  | -3.387 | -3.308 | -1.844 |
|  |  |  |  | (4.357) | (4.359) | (3.812) |
| Number of line workers | |  |  | 1.513 | 1.148 | -0.247 |
|  |  |  |  | (4.198) | (4.300) | (4.038) |
| Square of Number of line workers | | |  | -6.003^**^ | -6.286^**^ | -4.631 |
|  |  |  |  | (2.820) | (2.977) | (2.868) |
| Cube of Number of line workers | | |  | -5.151^**^ | -5.618^**^ | -2.739 |
|  |  |  |  | (2.433) | (2.553) | (2.578) |
| SAM |  |  |  |  | -0.099^*^ | -0.142^***^ |
|  |  |  |  |  | (0.055) | (0.046) |
| Work hours |  |  |  |  | -0.074 | -0.058 |
|  |  |  |  |  | (0.126) | (0.119) |
| Style - consecutive days in production | | |  |  | 0.014 | 0.011 |
|  |  |  |  |  | (0.016) | (0.015) |
| Tardiness (minutes) | |  |  |  |  | -0.003^*^ |
|  |  |  |  |  |  | (0.002) |
| Total absenteeism (%) | |  |  |  |  | -0.001 |
|  |  |  |  |  |  | (0.003) |
| Productivity (%) |  |  |  |  |  | 0.034^***^ |
|  |  |  |  |  |  | (0.005) |
| Observations | 4,191 | 4,191 | 4,191 | 4,191 | 4,191 | 4,191 |
| *Note:* | ^*^p<0.1; ^**^p<0.05; ^***^p<0.01 | | |  |  |  |
|  | Standard errors corrected for serial correlation using Newey-West variance estimator. | | | | | |

**S1R Table. Productivity-treatment-only OLS regressions with hourly wage as the dependent variable.**

|  | *Dependent variable:* | |  |  |  |  |
| --- | --- | --- | --- | --- | --- | --- |
|  | Hourly wage (baht) | |  |  |  |  |
|  | 1 | 2 | 3 | 4 | 5 | 6 |
| Productivity treatment | -0.643 | -0.343 | 0.172 | 0.03 | -0.12 | -0.085 |
|  | (1.208) | (1.235) | (0.981) | (0.993) | (1.002) | (1.005) |
| Overtime |  | 4.040^***^ | 4.085^***^ | 4.084^***^ | 4.095^***^ | 1.926^*^ |
|  |  | (0.689) | (0.663) | (0.646) | (0.647) | (1.131) |
| Familiarity |  |  | 0.050^***^ | 0.048^***^ | 0.048^***^ | 0.047^***^ |
|  |  |  | (0.008) | (0.008) | (0.008) | (0.008) |
| Quality Rate (%) | |  |  | 0.331^***^ | 0.336^***^ | 0.335^***^ |
|  |  |  |  | (0.074) | (0.075) | (0.075) |
| Number of line workers | |  |  | 16.964 | 17.814 | 16.938 |
|  |  |  |  | (12.790) | (12.652) | (12.844) |
| Square of Number of line workers | | |  | -16.135 | -15.248 | -15.233 |
|  |  |  |  | (11.597) | (11.264) | (11.222) |
| Cube of Number of line workers | | |  | -24.168^***^ | -22.992^***^ | -22.785^***^ |
|  |  |  |  | (8.617) | (8.224) | (8.204) |
| Work hours |  |  |  |  | 0.218^***^ | 0.217^***^ |
|  |  |  |  |  | (0.071) | (0.072) |
| SAM |  |  |  |  |  | 0.835^**^ |
|  |  |  |  |  |  | (0.366) |
| Style - consecutive days in production | | |  |  |  | 0.01 |
|  |  |  |  |  |  | (0.035) |
| Tardiness (minutes) | |  |  |  |  | -0.009 |
|  |  |  |  |  |  | (0.006) |
| Unplanned absenteeism (%) | | |  |  |  | -0.005 |
|  |  |  |  |  |  | (0.010) |
| Observations | 4,191 | 4,191 | 4,191 | 4,191 | 4,191 | 4,191 |
| *Note:* | ^*^p<0.1; ^**^p<0.05; ^***^p<0.01 | | |  |  |  |
|  | Standard errors corrected for serial correlation using Newey-West variance estimator. | | | | | |

**S1S Table**. **OLS regressions on profit-per-garment.**

|  | *Dependent variable:* | |  |  |  |  |
| --- | --- | --- | --- | --- | --- | --- |
|  | Factory Profit per Garment (baht) | | |  |  |  |
|  | 1 | 2 | 3 | 4 | 5 | 6 |
| Productivity treatment | -3.448^***^ | -3.519^***^ | -3.520^***^ | -3.374^***^ | -3.422^***^ | -3.432^***^ |
|  | (0.922) | (0.969) | (0.959) | (1.000) | (0.990) | (0.990) |
| PQWR treatment | -3.497^***^ | -3.533^***^ | -3.492^***^ | -3.204^***^ | -3.476^***^ | -3.408^***^ |
|  | (0.814) | (0.838) | (0.806) | (0.895) | (0.906) | (0.907) |
| Target wage treatment | -3.015^***^ | -3.049^***^ | -3.120^***^ | -3.114^***^ | -3.170^***^ | -3.177^***^ |
|  | (0.762) | (0.779) | (0.841) | (0.803) | (0.799) | (0.799) |
| Overtime |  | -0.518 | -0.514 | -0.484 | -0.531 | -0.596 |
|  |  | (0.533) | (0.529) | (0.528) | (0.541) | (0.994) |
| Familiarity |  |  | -0.003 | -0.003 | -0.003 | -0.003 |
|  |  |  | (0.008) | (0.008) | (0.009) | (0.009) |
| Number of line workers | |  |  | 8.139 | 3.853 | 4.817 |
|  |  |  |  | (6.361) | (6.634) | (6.805) |
| Square of Number of line workers | | |  | 7.693^***^ | 7.756^***^ | 7.346^***^ |
|  |  |  |  | (2.418) | (2.629) | (2.578) |
| Cube of Number of line workers | | |  | 4.952 | 5.753^*^ | 5.455 |
|  |  |  |  | (3.523) | (3.371) | (3.444) |
| SAM |  |  |  |  | -1.064^***^ | -1.058^***^ |
|  |  |  |  |  | (0.128) | (0.129) |
| Work hours |  |  |  |  |  | 0.016 |
|  |  |  |  |  |  | (0.339) |
| Style - consecutive days in production | | |  |  |  | -0.017 |
|  |  |  |  |  |  | (0.024) |
| Tardiness (minutes) | |  |  |  |  | 0.012^*^ |
|  |  |  |  |  |  | (0.006) |
| Unplanned absenteeism (%) | | |  |  |  | 0.047 |
|  |  |  |  |  |  | (0.040) |
| Observations | 1,692 | 1,692 | 1,692 | 1,692 | 1,692 | 1,692 |
| *Note:* | ^*^p<0.1; ^**^p<0.05; ^***^p<0.01 | | |  |  |  |
|  | Standard errors corrected for serial correlation using Newey-West variance estimator. | | | | | |
